# Incidence, Survival, and Mortality Trends of Cancers Diagnosed in Adolescents and Young Adults (15–39 Years): A Population-Based Study in The Netherlands 1990–2016

**DOI:** 10.3390/cancers12113421

**Published:** 2020-11-18

**Authors:** Daniël J. van der Meer, Henrike E. Karim-Kos, Marianne van der Mark, Katja K. H. Aben, Rhodé M. Bijlsma, Anita W. Rijneveld, Winette T. A. van der Graaf, Olga Husson

**Affiliations:** 1 Department of Psychosocial Research and Epidemiology (PSOE), Netherlands Cancer Institute (NKI), 1066 CX Amsterdam, The Netherlands; d.van.der.meer@nki.nl; 2 Netherlands Comprehensive Cancer Organisation (IKNL), 3511 DT Utrecht, The Netherlands; H.Karim-Kos@iknl.nl (H.E.K.-K.); M.vanderMark@iknl.nl (M.v.d.M.); k.aben@iknl.nl (K.K.H.A.); 3 Department of Public Health, Erasmus MC University Medical Center, 3015 GD Rotterdam, The Netherlands; 4 Radboud Institute for Health Sciences (RIHS), Radboud University Medical Center, 6525 EZ Nijmegen, The Netherlands; 5 Department of Medical Oncology, University Medical Center Utrecht, 3584 CX Utrecht, The Netherlands; R.M.Bijlsma@umcutrecht.nl; 6 Department of Hematology, Erasmus MC Cancer Center, 3015 GD Rotterdam, The Netherlands; a.rijneveld@erasmusmc.nl; 7 Department of Medical Oncology, Netherlands Cancer Institute—Antoni van Leeuwenhoek Hospital, 1066 CX Amsterdam, The Netherlands; w.vd.graaf@nki.nl; 8 Department of Medical Oncology, Radboud University Medical Center, 6525 EZ Nijmegen, The Netherlands; 9 Division of Clinical Studies, Institute of Cancer Research, London SM2 5NG, UK

**Keywords:** adolescents and young adults, AYA, incidence, survival, mortality, cancer epidemiology, cancer trends, The Netherlands

## Abstract

**Simple Summary:**

Adolescents and young adults (AYAs, aged 15–39 years) with cancer form a distinct patient population within the oncology care setting that is often overlooked in favour of paediatric and older adult patients. As such, specific knowledge on AYAs and their distinct spectrum of cancers is limited. Worldwide, cancer is increasing and it is among the major causes of death among AYAs. Cancer prognosis among AYAs has also been shown to lag behind that of younger and older patients suffering from similar diseases. To address these problems, better understanding of AYA cancers is needed. This study aims to provide an overview of the specific cancer trends among AYAs and the changes that have occurred in the Netherlands since 1990 in terms of incidence, survival, and mortality. This information will provide a solid foundation from which to guide future studies upon, aimed at acquiring more detailed cancer knowledge within the AYA domain.

**Abstract:**

Adolescent and young adult (AYA) cancer patients, aged 15–39 years at primary cancer diagnosis, form a distinct, understudied, and underserved group in cancer care. This study aimed to assess long-term trends in incidence, survival, and mortality of AYA cancer patients within the Netherlands. Data on all malignant AYA tumours diagnosed between 1990–2016 (*n* = 95,228) were obtained from the Netherlands Cancer Registry. European age-standardised incidence and mortality rates with average annual percentage change (AAPC) statistics and five-year relative survival rates were calculated. The overall cancer incidence increased from 54.6 to 70.3 per 100,000 person-years (AAPC: +1.37%) between 1990–2016, and increased for both sexes individually and for most cancer types. Five-year relative survival overall improved from 73.7% in 1990–1999 to 86.4% in 2010–2016 and improved for both sexes and most cancer types. Survival remained poor (<60%) for rhabdomyosarcoma, lung, stomach, liver, bladder, and pancreatic carcinomas, among others. Mortality rates among male AYAs overall declined from 10.8 to 6.6 (AAPC: −1.64%) and from 14.4 to 10.1 per 100,000 person-years (AAPC: −1.81%) for female AYAs since 1990. Mortality rates remained unchanged for male AYAs aged 20–24 and 25–29 years. In conclusion, over the past three decades, there has been a considerable increase in cancer incidence among AYAs in the Netherlands. Meanwhile, the survival improved and the mortality overall declined. Survival at five-years now well exceeds above 80%, but did not do so for all cancer types.

## 1. Introduction

Worldwide, adolescents and young adults (AYA) form a distinct, understudied, and underserved group within the oncology domain that is often overlooked [1,2,3,4]. In the Netherlands care for children with cancer is centralised based on their age, 0–17 years at diagnosis, whereas patients 18 years or older are being treated in any hospital in the Netherlands and centralisation only takes place based on certain tumour types. However, in practice this distinction is less clear, as was illustrated by a recent Dutch study, which observed that children before the age of 18 and diagnosed with (young) adult type tumours (e.g., chronic myeloid leukaemia, thyroid carcinomas, and melanoma and skin carcinomas) are less often referred for treatment to a specialised paediatric oncology centre [5]. To improve this, the nationwide AYA “young & cancer” care network (https://ayazorgnetwerk.nl) was established in 2016 and has made it their main mission to raise national awareness and to optimise the quality and access to care received by AYAs with cancer in the Netherlands. Nevertheless, care for the AYA age group is challenging at a global level due to low numbers and specific age adjusted care needs beyond the primary cancer diagnosis.

The definition of AYA for cancer purposes has evolved over time. In 2006, a broad age range of 15–39 years was proposed by the United States (US) National Cancer Institute (NCI) with support from the LIVESTRONG Foundation [1], which has been adopted by The European Network for Cancer in Children and Adolescents.

AYA cancers form a unique spectrum of malignancies comprised of the tail end of cancers frequently found in children (e.g., acute lymphatic leukaemia, Ewing sarcoma), cancers that primarily affect older adults (e.g., colorectal, lung, and breast carcinomas) and cancers specific to the AYA population (e.g., Hodgkin lymphoma, melanoma, germ cell tumours, and thyroid carcinomas). Although rare, cancer at AYA age is 4–6 times more frequently diagnosed when compared to cancers diagnosed in children during the first 15 years of life [6,7]. Each year, an estimated 66,000 AYAs will develop cancer in Europe alone, representing 4% of all invasive cancer diagnoses [6,7]. The incidence of AYA cancers is increasing in most countries worldwide, including the US and United Kingdom, with sharp increases in incidence observed for thyroid, testicular, and obesity-related cancers (e.g., colorectal, uterus, pancreas, gallbladder, and liver) [8,9].

Cancer is responsible for approximately 25% of all deaths at AYA age, making it the leading cause of disease-related death in this population in high-income countries. Still, improvements in the survival rate of several cancer types among AYAs are encouraging and overall survival rates now well exceed 80% at five-years of follow-up [10,11]. Previously, it was shown that gains in survival among AYAs lagged behind those found in children (aged < 15 years) and older adults (aged ≥ 39 years) [12,13], but according to a recent study (1988–2014), this gap has been closed [14]. Nevertheless, there is still much outcome heterogeneity between the various cancer types among AYAs, with the prognosis of some still lagging considerably behind those found in paediatric and adult patients.

The societal and economic impact of cancer at AYA age is great given the major effects of premature morbidity and mortality [15]. To improve cancer-associated outcomes for AYAs, the NCI and LIVESTRONG Foundation released a report in 2006 on AYA oncology entitled “Closing the gap” [1]. An update on the scientific gaps and opportunities for future research on AYAs was provided in a 2013 workshop entitled “Next Steps for Adolescents and Young Adult Oncology: A Scientific Update” [7]. To guide future research efforts, both workshops highlighted the importance of continuing to monitor cancer trends and recommended the examination of the unique incidence and survival trends of cancer in general and for individual cancer types by sex, age, and other subgroups within the AYA population when sample size allows [1,7]. This study aimed to assess long-term trends in incidence, survival, and mortality of AYA cancer in the Netherlands between 1990–2016 for the total population and by sex, age group and cancer type.

## 2. Materials and Methods

### 2.1. Data Sources

Data on primary cancer incidence and survival were obtained from the nationwide population-based Netherlands Cancer Registry (NCR), which includes data on all newly diagnosed cancer cases in the Netherlands since 1989 with exception of basal cell carcinomas of the skin. The NCR is hosted by the Netherlands Comprehensive Cancer Organisation (IKNL) and is based on notification of all newly diagnosed malignancies in the Netherlands by the Automated Pathological Archive (PALGA), supplemented by notifications from the Dutch hospital database and various haematology laboratories. Following notification, trained registrars collect relevant information on patient and tumour characteristics from the medical records 6–12 months after diagnosis. All cancers included in the NCR are coded using the International Classification of Diseases for Oncology (ICD-O). Cancers diagnosed before 1993 were coded using the first edition of the ICD-O. Between 1993 and 2000, the second edition of the ICD-O was used and since 2001, the ICD-O third edition [16]. Information on vital status (e.g., alive, dead) and emigration was obtained by annual linkage of the NCR with the nationwide Personal Records Database (BRP; Basisregistratie personen) that holds vital statistics on all residents in the Netherlands. Mortality data on malignant cancer (ICD-10 code C00-C97) for the period 1990–2016 were obtained from the cause-of-death statistics of Statistics Netherlands (http://statline.cbs.nl).

### 2.2. Data Selection

All malignant neoplasms (ICD-O-3 behaviour code /3) and pilocytic astrocytoma’s (ICD-O-3 M9421, behaviour code /1) in patients aged 15–39 years and diagnosed between 1990 and 2016 were extracted from the NCR. From this selection, we excluded low-grade chondrosarcomas, myelodysplastic syndromes, Langerhans cell tumours, borderline ovarian tumours and carcinomas of the appendix, as these groups were not consistently coded as malignant tumours throughout the entire study period. All malignant neoplasms were classified according to the Surveillance, Epidemiology, and End Results Program (SEER) AYA site recode classification scheme, adapted from the classification scheme proposed by Barr et al. [17]. Additionally, the international rules concerning multiple cancers were applied to allow comparison of the results with other studies [18].

### 2.3. Statistical Analyses

Descriptive analyses of the average number of new cases per year and cancer type distribution, including all main AYA cancer sites and carcinomas, were performed.

#### 2.3.1. Age Standardised Rates

Annual and period specific incidence and mortality rates for the period 1990–2016 were calculated per 100,000 person-years using the mid-year population size obtained from Statistics Netherlands (http://statline.cbs.nl). Rates were age-standardised with weights based on the five-year age groups from the 1976 European standard population [19].

#### 2.3.2. Joinpoint Regression Analyses

Changes in incidence and mortality over the entire study period 1990–2016 were evaluated with joinpoint regression analyses by calculating average annual percentage change (AAPC) statistics and corresponding 95% confidence intervals (CIs) [20]. All age-standardised incidence and mortality rates and AAPC estimates were calculated using the freely available Joinpoint Regression Program version 4.8.0.1 [21]. All models were plotted using the “grid search method” and the “uncorrelated error model”. The number of points to place between adjacent observed x-values in the grid search method was set at 3 (0 at default). Correlation of the random errors in the data were analysed by repeating the analyses multiple times, each time with a different value of the autocorrelation parameter (0.1, 0.2, and 0.3). Outcomes were similar to those obtained with the uncorrelated error model, which was then selected for the final analyses, as correcting for autocorrelation can reduce the power to detect joinpoints. A minimum of zero and a maximum of five joinpoints was allowed. Selection of the final model was done using the recommended Bayesian information criteria 3. Further parameters within the joinpoint program were kept at their default setting.

#### 2.3.3. Relative Survival

Relative survival was used as estimator for disease-specific survival and is the ratio of the observed survival in cancer patients by the expected survival in a comparable general population matched by sex, age, and calendar year. Expected survival lifetables of the Dutch general population were retrieved from Statistics Netherlands and contained annual survival probability data for both sexes aged 0–99 years between 1950–2019. Five-year relative survival estimates were determined using the Ederer II method and were computed using the -Strs- Stata command developed by Dickman and colleagues [22]. End of follow-up was defined as date of death, date of emigration, or 1 February 2019, whichever came first. Survival trends over time were assessed by modelling the relative survival using a generalised linear model and assuming a Poisson distribution for the observed number of deaths [23]. Significance of linear trends, were obtained with *p*-values from a likelihood ratio test comparing a model including the midpoint of the calendar period and a model without calendar period. A *p*-value < 0.05 was considered to be statistically significant.

Analyses were performed with Stata version 16.1. Outcomes of the various analyses are presented for all AYAs combined and according to age group (15–19, 20–24, 25–29, 30–34, and 35–39 years), sex (male, female), calendar period (1990–1999, 2000–2009, 2010–2016, and 1990–2016), and cancer type whenever feasible. The study was approved by the Privacy Review Board of the NCR. The data used in this study can be reasonably requested from the NCR (study number K16.305, www.iknl.nl).

## 3. Results

### 3.1. Study Population

Table 1 shows the total and average number of new cancer cases, incidence rates and AAPCs by sex, age, cancer type and calendar period. In total, 95,228 primary AYA cancers were registered in the Netherlands between 1990–2016. More than half were diagnosed among females (59.0%, *n* = 56,190). The total number of AYA cases diagnosed with cancer increased substantially with age from 5.7% (*n* = 5434) in AYAs aged 15–19 years to 41.8% (*n* = 39,798) in AYAs aged 35–39 years (Table 1).

### 3.2. Cancer Type Distribution

**Figure 1:**
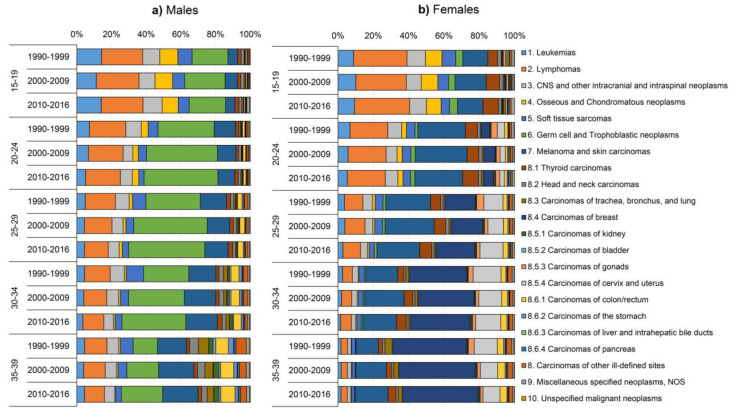
Relative frequencies (%) of cancer types according to the adolescent and young adult (AYA) site recode classification scheme by age group and calendar period for (**a**) male and (**b**) female AYA cancer patients diagnosed at age 15–39 years in the Netherlands between 2010–2016.

Figure 1 displays the distribution of AYA cancers stratified by sex, age and calendar period. The data underlying this figure are included in Table 1 and Table S1. The cancer type distribution among AYAs differed considerably between sex and age groups. Specific for male AYAs was the large contribution of germ cell tumours for all age groups, peaking at 44.0% in the 25–29 age group in 2010–2016. The contribution of lymphomas decreased with age, whereas carcinomas and “melanoma and skin carcinomas” became more prominent, increasing up to 28.0% and 20.4%, respectively, in the 35–39 age group in 2010–2016. Of all carcinomas, most frequently diagnosed among males were colorectal and lung carcinomas, contributing up to 8.3% and 3.8% in 2010–2016 at age 35–39, respectively (Figure 1a). Among female AYAs, lymphomas had the largest contribution in the 15–19 age group (31.2% in 2010–2016), whereas carcinomas dominated the subsequent age groups, contributing up to 70.6% in the 35–39 age group in 2010–2016. Of all carcinomas diagnosed in female AYAs, thyroid carcinomas contributed most in the 15–19 and 20–24 years age groups (8.8% and 8.9%, respectively in 2010–2016). Carcinomas of the breast, cervix, and uterus dominated the older age groups, comprising up to 43.0% (breast) and 9.7% (cervix/uterus) of all cancers diagnosed among female AYAs aged 35–39 years in 2010–2016 (Figure 1b). Time-related changes in the distribution of AYA cancers were overall less pronounced (≤5% between 1990–1999 and 2010–2016) for the majority of cancer types. Exception to this were the ≥10% increases in the distribution of germ cell and trophoblastic neoplasms among male AYAs aged older than 15–19 years.

### 3.3. Trends in Incidence

**Figure 2:**
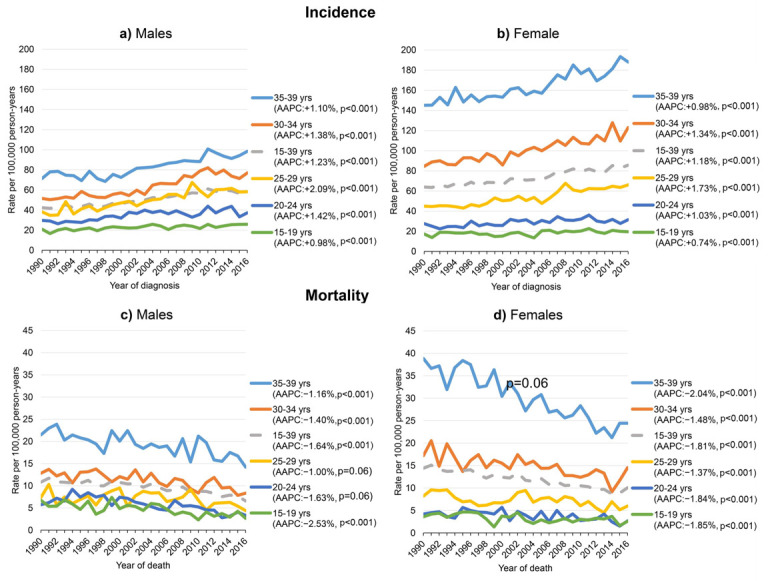
Age-standardised incidence and mortality rates per 100,000 person-years for (**a,c**) male and (**b**,**d**) female adolescents and young adults (AYAs) diagnosed with or passed away due to cancer in the Netherlands between 1990–2016. Rates were standardised using weights from the 1976 European standard population. Note the non-significant average annual percentage change (AAPC) estimates in panel c for male AYA cancer patients aged 20–24 and 25–29 years.

Overall, the incidence of AYA cancers increased significantly from 54.6 in 1990–1999 to 70.3 per 100,000 person-years in 2010–2016 (AAPC: +1.37%) and likewise increased significantly over time for both sexes and for all age groups. Incidence rates were overall higher for female AYAs with around 22 per 100,000 person-years in each calendar period (1990–1999, 2000–2009, and 2010–2016). Rates were higher for male AYAs aged 15–19 and 20–24 years. From age 25–29 years onward, rates became increasingly higher for female AYAs in each calendar period (Table 1, Tables S1 and S2, and Figure 2). Increases in incidence over time were similar for both sexes for all ages combined (AAPC: +1.23% in males; AAPC: +1.18% in females). In both sexes, the highest increases in incidence were observed in the 25–29 age group (AAPC: +2.09% in males; AAPC: +1.73% in females), whereas they were the lowest in the 15–19 age group (AAPC: +0.98% in males; AAPC: +0.74% in females) (Tables S1 and S2).

For the various cancer types, most marked increases in incidence since 1990 were observed for AYAs with “unspecified intracranial and intraspinal neoplasms” (AAPC: +2.58% in males; AAPC: +3.00% in females), melanomas (AAPC: +2.30% in males; AAPC: +1.89% in females), skin carcinomas (AAPC: +2.60% in males; AAPC: +2.77% in females) and carcinomas of the thyroid (AAPC: +2.56% in males; AAPC: +3.55% in females), kidney (AAPC: +2.88% in males; AAPC: +3.01% in females) and colorectum (AAPC: +1.88% in males; AAPC: +1.83% in females) (Table 1, Tables S1 and S2). Significant rising trends exclusive to male AYAs were seen for chronic myeloid leukaemia’s (CML, AAPC: +2.24%), “gonadal germ cell and trophoblastic neoplasms” (AAPC: +3.73%), and miscellaneous specified neoplasms (AAPC: +2.32%). Increases in the incidence of “other and unspecified leukaemia’s” (AAPC: +2.38%), chondrosarcomas (AAPC: +2.32%), “non-gonadal germ cell and trophoblastic neoplasms” (AAPC: +1.85%) and liver (AAPC: +2.54%) and pancreas carcinomas (AAPC: +2.41%) were seen in female AYAs only (Table 1, Tables S1 and S2). Significant declines in incidence were observed among male AYAs with Kaposi sarcomas (AAPC: −8.42%), fibromatous neoplasms (AAPC: −2.74%) and carcinomas of other ill-defined sites (AAPC: −4.06%), while carcinomas of the gonads (AAPC: −2.35) and lung (AAPC: −1.17) significantly declined in females only (Table S2).

### 3.4. Trends in Survival

**Figure 3:**
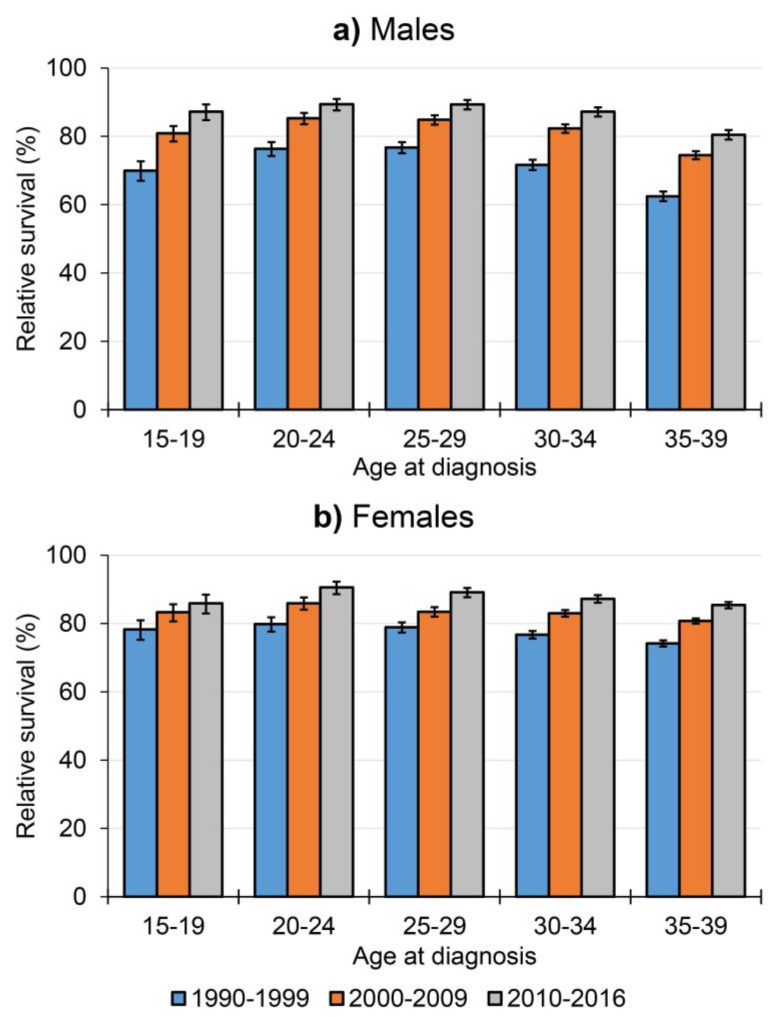
Five-year relative survival outcomes with 95% confidence intervals over time for (**a**) male and (**b**) female adolescent and young adult (AYA) cancer patients by age group in the Netherlands between 1990–2016.

The five-year relative survival of all AYAs combined improved significantly over time from 73.7% (95%CI: 73.2, 74.1) in 1990–1999 to 86.4% (95%CI: 86.0, 86.9) in 2010–2016. Survival gains were more prominent in male compared to female AYAs, increasing from 70.0% (95%CI: 69.2, 70.8) to 85.7% (95%CI: 85.0, 86.4) for males (15.8% increase) and from 76.2% (95%CI: 75.6, 76.8) to 86.9% (95%CI: 86.3, 87.5) for female AYAs (10.7% increase) from 1990–1999 to 2010–2016 (Table 2, Tables S3 and S4, and Figure 3). Survival outcomes in 2010–2016 were similarly high for most age groups around 87–90% at five-years of follow-up for both sexes, but lagged somewhat behind in male AYAs aged 35–39 years (80.5% [95%CI: 79.1, 81.9]) (Table 2, Tables S3 and S4, and Figure 3).

**Figure 4:**
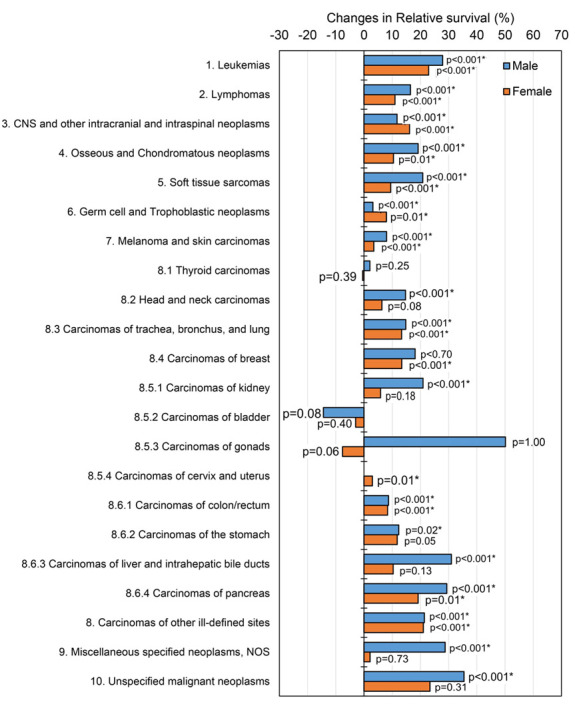
Cancer type specific changes (%) in five-year relative survival outcomes between 1990–1999 and 2010–2016 for male and female adolescent and young adult (AYA) cancer patients diagnosed in the Netherlands between 1990–2016. *p*-values were obtained from a likelihood ratio test comparing a model including the midpoint of the calendar period and a model without calendar period. A *p*-value <0.05 was considered to be statistically significant (denoted with *).

Among the various cancer types, largest improvements (>30%) in survival since 1990–1999 were observed for Kaposi sarcoma (66.5% and 55.0% increase in males and females, respectively) and “unspecified intracranial and intraspinal neoplasms” (42.2% and 30.6% increase in males and females, respectively). Large improvements in survival exclusive to male AYAs were observed for CML (44.1% increase up to 95.7%), unspecified malignant neoplasms (35.5% increase up to 51.6%) and liver carcinomas (31.0% increase up to 41.6%), whereas this was the case for Ewing sarcomas (33.0% increase up to 66.3%) and acute lymphatic leukaemia’s (ALL, 30.6% increase up to 75.2%) for female AYAs only. Improvements in survival were overall more prominent among male AYAs for most cancer types (Figure 4). A significant 10.3% (*p*-value = 0.04) decline in bladder carcinoma survival for both sexes combined was observed between 1990–1999 and 2010–2016, but was not significantly shown in males and females individually (Table 2, Tables S3 and S4, and Figure 4). For several cancer types, non-significant time-related changes in survival were observed, most prominently among females (nine groups more compared to males) (Table 2, Tables S3 and S4).

Favourably high survival outcomes (>90%) in 2010–2016 were observed for all AYAs with Hodgkin lymphomas, “gonadal germ cell and trophoblastic neoplasms”, “melanoma and skin carcinomas”, fibromatous neoplasms, Kaposi sarcomas, and thyroid carcinomas. For male AYAs specific, survival was also favourably high for CMLs, chondrosarcomas, and for carcinomas of the breast and gonads (which likely relates to low case numbers), whereas survival was high for ependymomas and “non-gonadal germ cell and trophoblastic neoplasms” in female AYAs only. For all AYAs, survival remained poor (<60%) for rhabdomyosarcoma, unspecified soft tissue sarcomas, unspecified malignant neoplasms, and carcinomas of the lung, stomach, liver, pancreas, bladder and other ill-defined sites. In males, the survival was also particularly low for astrocytoma’s, Ewing sarcomas, and other specified intracranial and intraspinal neoplasms (Table 2, Tables S3 and S4).

### 3.5. Trends in Mortality

In total, 16,931 AYA cancer mortality cases were registered in the Netherlands between 1990–2016 (Table S5). Cancer mortality rates were generally higher among female AYAs. This was especially the case for the 30–34 and 35–39 age groups, whereas the mortality was relatively higher for male AYAs aged 15–19 and 20–24 years (Figure 2). Mortality rates for all female AYAs combined significantly declined from 14.4 to 10.1 per 100,000 person-years (AAPC: −1.81%) between 1990–2016, whereas rates overall declined from 10.8 to 6.6 per 100,000 person-years (AAPC: −1.64%) in males (Figure 2). Age-specific mortality rates among female AYAs significantly declined for all age groups since 1990. Non-significant declines in mortality were observed for male AYAs 20–24 and 25–29 years of age, but significantly declined otherwise (Figure 2).

## 4. Discussion

This study provided a comprehensive overview of long-term trends in incidence, survival and mortality among all AYA cancer patients aged 15–39 years and diagnosed in the Netherlands from 1990 through 2016. In general, the incidence of AYA cancer has increased. Meanwhile, the survival improved and the mortality declined over time for both sexes and for all age groups. Our findings further highlight a changing pattern in the distribution of cancer types based on AYA sex, age, and calendar period. Although progress among AYAs has been encouraging for most cancer types, survival gains and outcomes at five-years of follow-up remained poor (<60%) for AYAs with rhabdomyosarcoma, unspecified soft tissue sarcomas, unspecified malignant neoplasms and carcinomas of the lung, bladder, stomach, liver, pancreas, and other ill-defined sites.

### 4.1. Cancer Type Distribution

It is well-known that the spectrum of cancers in AYAs is distinct and differs considerably from that in paediatric and older adult cancer patients [10,15,24,25]. Our data reconfirm and add to this by also presenting the distribution of AYA cancers over time, which highlights the minimal impact that time has had on the distribution of AYA cancers from 1990–1999 to 2010–2016. Our findings further emphasise the importance of distinguishing AYAs based on sex and age instead of grouping them all together for analytical purposes as the distribution of AYA cancers differs substantially based on these factors.

### 4.2. Trends in Incidence

Similar to earlier findings in most countries worldwide, the incidence of AYA cancers in the Netherlands has increased significantly since 1990 [7,8,9,24,25]. Rising trends in overall cancer incidence among AYAs have been attributed to thyroid cancer overdiagnosis (detection of cancers that would not have affected the lives of individuals during their lifetimes when left undetected) and trends stabilised after these malignancies were excluded from the analyses, in particular for females [25,26]. In our study, stabilisation of trends only occurred for female AYAs aged 20–24 years (AAPC: +0.53, *p* = 0.20) after excluding thyroid carcinomas from the analyses. This indicates that thyroid cancer alone does not provide a full explanation for the current rise in cancer incidence among AYAs in the Netherlands.

Another reason may be the increasing number of children (aged 0–17 years) diagnosed with cancer in the Netherlands (incidence increased annually with 0.6% between 1990–2017) [27]. Survival of childhood cancers is high (>80% at five-years of follow-up) and survivors have been shown to have a higher risk of developing subsequent malignancies when compared with the general population, including cancers typically seen in AYAs (e.g., bone and soft tissue sarcomas and breast, thyroids, and skin carcinomas) [28]. In our study, we have included all primary cancers that were diagnosed at AYA age, including subsequent malignancies of those that were first diagnosed with cancer during childhood. As such, the higher subsequent cancer risk of childhood cancer survivors likely contributes to some extent to the rise in cancer incidence at AYA age, but we were unable to test this.

The relative higher cancer incidence rates among female AYAs have been observed worldwide and could be explained by the extension of the upper-age-limit of AYAs from 25 to 39 years and is mainly based on the increased incidence of so-called female cancers (i.e., breast, cervix, and uterus) [7,15,24,25]. The approximately three times higher rates of thyroid carcinoma incidence among females likely also contributed.

#### 4.2.1. Melanoma and Skin Carcinomas

The relative high incidence rates (approximately two times higher in females) and the noticeable increases in melanomas and skin carcinomas among all AYAs over time likely result from increased sun exposure and use of tanning devices, but could also reflect increased cancer awareness [29,30]. The extent to which these factors apply to the youngest AYAs could be argued, as exposure time in this population is limited. However, greater use and earlier age at first sunbed use have been associated with increased melanoma risk (76% of melanomas were attributed to sunbed use at age 18–29 years) in previous research [29]. It was also suggested that younger people might be more biologically susceptible to the carcinogenic effects of artificial ultraviolet radiation [29]. Support for this was provided in a more recent study, were mutations associated with ultraviolet radiation damage were found at similar rates among AYAs compared with older patients [31]. Primary prevention efforts aimed at reducing ultraviolet ray exposure have already proven effective in lowering melanoma incidence among Australian (1998–2012) and American AYAs (2007–2016) and might also do so in other countries, especially among those with high sunbed use [25,30].

#### 4.2.2. Thyroid and Kidney Carcinomas

In line with previous global finding, rising trends in incidence were among the highest for AYAs diagnosed with thyroid and kidney carcinomas and a markedly higher burden of thyroid carcinomas was observed within the female AYA population [8,9,15,24,25]. These rising trends have been primarily attributed to overdiagnosis [9,32,33]. There is also some evidence suggesting that exposure to ionizing radiation (particularly in childhood) could influence the incidence of thyroid carcinomas in young adults [34]. Still, overdiagnosis does not seem to provide a full explanation, as mortality rates of thyroid carcinomas in the US are steadily increasing, likely due to a true rise in advanced stage papillary thyroid cancer (1974–2013) [35]. It also remains unclear what exactly causes the higher thyroid cancer burden among females [36]. Gaining increased genomic and proteomic knowledge is warranted, as it may provide a solid basis to better understand the gender disparities within the AYA cancer domain.

#### 4.2.3. Colorectal, Liver, and Pancreas Carcinomas

Worldwide, the AYA population has seen a distinct rise in incidence of colorectal carcinomas (CRC) [8,37,38]. Rates in the general population have either been stabilizing or are decreasing in high-income countries due to screening practices [39,40]. It is currently not clear what causes the increase among AYAs, but it could be owing to the increased use of colonoscopy among symptomatic cases and advances in imaging and pathological techniques [40]. Environmental factors (e.g., Western diet, smoking, and alcohol consumption) and heredity predisposition (e.g., Lynch syndrome, familial adenomatous polyposis) have also been implicated, although most AYA CRCs were found to be sporadic in nature [41,42,43]. It has been hypothesised that AYA CRCs have a unique biology relative to those arising at older ages, but contemporary knowledge is limited [43,44]. The rising levels of obesity might also drive some of the burden. Obesity is a well-known risk factor associated with CRC and has also been proposed to be partly responsible for the increase in liver and pancreas carcinomas, here observed exclusively among female AYAs [45,46].

#### 4.2.4. Gonadal Germ Cell Tumours

Specific to male AYAs was the high burden and prominent increase in gonadal germ cell tumours (testis) since 1990. These findings are in line with those observed globally and highlight the large contribution that these tumours have on the overall rising trend in incidence of cancer among males; in fact, this group is the main explanation for this observation [8,25,47]. Although the aetiology of these cancers in the AYA population is poorly understood, an association was found between higher levels of neonatal androgens and increased testis cancer risk among adolescents (15–19 years), but not infants (0–4 years) [48]. Exposure to certain persistent organochlorine pesticides during foetal life might also increase the risk of testicular germ cell tumours [49].

#### 4.2.5. Breast Carcinomas

Among female AYAs, the incidence of breast cancer steadily increased since 1990, but it is not well understood what exactly drives these trends. For the older AYA age groups, the well-known lifestyle factors often associated with increased breast cancer risk (e.g., early age at menarche, late age at first childbearing, low number of pregnancies, not breast feeding, and exogenous hormone use) are likely responsible in part for the increase [50]. In younger AYAs, genetic predisposition (BRCA 1/2) is likely an important contributing risk factor for breast cancer, but also excessive alcohol consumption and smoking habits [50]. In a 2006 meta-analysis, high breast density was found to be a strong risk factor for breast cancer at all ages and likely also drives some of the burden [51].

#### 4.2.6. Lung Carcinomas

Similar to other studies, an unexplained decline in lung carcinoma incidence among female AYAs was found [8,9,26]. In contrast to existing literature, no declining trend was observed for male AYAs, whereas this was shown in previous research in various countries worldwide (e.g., US, Canada, Spain, etc.) between 1998–2012 [8]. However, the same paper did not observe any declines in lung carcinomas among AYAs in the Netherlands [8]. Declines likely relate in part to the decreased rate of tobacco smoking due to successful prevention campaigns, but considering the young age of the AYA population, the impact of smoking in causing the disease is likely limited [9]. Moreover, there is a relatively high percentage of AYAs with lung cancer who never smoked and have driver mutations, such as EGFR, ALK, KRAS, and ROS1 [9,52,53]. Future research should attempt to find more likely explanatory factors underlying these declining trends among AYAs and in general [9].

#### 4.2.7. Kaposi Sarcoma and Non-Hodgkin Lymphoma

Historically, the human immunodeficiency virus (HIV)/acquired immune deficiency syndrome (AIDS) epidemic in the 1980s to early 1990s lead to a marked increase in Kaposi sarcoma and non-hodgkin lymphoma (NHL) incidence, especially among male AYAs. After the epidemic subsided (due to improved antiretroviral treatment and HIV control), the incidence started stabilizing and declining for all age groups, albeit much slower in AYAs [9,54]. Based on this and other studies, the post-epidemic declines have maintained [8,9,10]. The large AAPC effect sizes for Kaposi sarcoma in this study are most likely affected by the small number of cases and subsequent incidence rates (*n* = 690 between 1990–2016).

### 4.3. Trends in Survival

Favourably high survival outcomes and promising gains among AYA cancer patients have been reported by multiple studies worldwide and now often exceed well above 80% (here 86.4% in 2010–2016) at five-years of follow-up [12,24,25,55]. As noted in a recent study, AYAs now demonstrate survival gains that are at least equal to those found in paediatric and older adult patient populations and it was concluded that the AYA survival gap has been closed. However, this was not the case for all malignancies and disparities were reportedly increasing between various AYA subsets (e.g., stage, race, ethnicity, etc.) [7,12,14]. Likewise, reported gains in 10-, 20-, and 25-year overall survival among five-year American AYA cancer survivors (treated between 1970–2005) remained overall inferior to that of the general population [55]. Survival outcomes among AYAs might in fact also be less favourable than those reported in the literature due to being artificially inflated by thyroid carcinoma overdiagnosis (five-year survival approaches 100%). However, after correcting for this and for the HIV/AIDS epidemic, Bleyer et al. reported that the overall relative survival still improved and exceeded 80% at five-years of follow-up in females and 75% in males based on data from 1975 through 2007 [9]. Consistent with these findings, exclusion of thyroid carcinomas from the survival analysis in this study also had no apparent effect and the observed relative survival outcomes and gains among AYAs remained favourably high.

#### 4.3.1. Kaposi Sarcoma and Non-Hodgkin Lymphoma

As in previous studies, survival outcomes in the past (1990–1999) were around 6% higher in female AYAs, but have become approximately equal between both sexes in 2010–2016 [12,14,55]. Lower survival outcomes among male AYAs in the past have been attributed to the increased burden of cancers related to the HIV/AIDS epidemic, which mainly affected males and historically had among the lowest survival outcomes (Kaposi sarcoma) [9]. The HIV/AIDS epidemic, however, does not appear to have negatively impacted the cancer survival outcomes in this study, as exemplified by the apparent lack in outcome changes when excluding Kaposi sarcoma and NHL from the analyses. This is not surprising considering that the prognosis of HIV/AIDS related cancers has drastically improved following the development of more effective treatment options, including targeted anti-CD20 immunotherapy [9,54]. This likely also resulted in the major improvements in survival of Kaposi sarcoma and HIV-related NHL, now reaching five-year relative survival outcomes well-above 85% among all AYAs combined in 2010–2016, whereas in the past, they were usually fatal [9,54]. Similar conclusions have been drawn elsewhere [14].

#### 4.3.2. Gonadal Germ Cell Tumours

More likely related to the catching up of the survival among male AYAs in this study is the increasing presence of gonadal germ cell tumours (testis) with their good prognostic outcomes (>95%). This is evidenced by the exclusion of these tumours from the analyses, which drastically lowered the survival outcomes among males with around 6% in 2010–2016, whereas the survival in females remained unaffected. Although a major driver, the increase in gonadal germ cell tumours does not provide a full explanation for the entire extent of improvements. Higher survival gains over time among male AYAs were also observed for most other cancers including common types, such as leukaemia’s, lymphomas, “melanoma and skin carcinomas”, and nearly all carcinomas, most of which had better survival outcomes in female AYAs in the past, but now for most cases have become equal for both sexes. Advances in diagnostic and treatment practices, therefore, likely also contributed.

Compared to the other sex and age groups, the five-year relative survival is still lower in male AYAs aged 35–39 years. This likely relates to the increased presence of carcinomas with increasing age, which overall had a much better prognosis in female AYAs, likely due to the high survival outcomes (>80%) of female cancers (i.e., breast, uterus, and cervix cancer), which are most commonly found in females aged 35–39 years. Recent literature also points to other factors responsible for the survival disparities between AYA subgroups, including stage, race, and social economic status, but these were not included in this study [14].

#### 4.3.3. Bladder Carcinomas

Whereas the survival improved for most cancers at AYA age, there was a noticeable decline in bladder carcinoma survival, although this was only significant for both sexes combined, likely due to low case numbers when stratified by sex. It is unclear what exactly causes this decline or the poor prognosis, as studies on bladder cancer at AYA age are sparse and often small in size [56,57]. Compared to older patients, AYA bladder carcinoma patients were shown to have better cause-specific and overall cancer survival outcomes and were more often diagnosed with low grade (75.5% vs. 48.6%) and localised stage disease (71.6% vs. 63.6%) [58]. Other studies reported similar findings [56,57]. Among AYAs, survival outcomes were only lower for non-Hispanic African Americans with low socioeconomic status compared with older patients [58]. However, this provides no explanation for the consistent decline in bladder cancer survival since 1990–1999. Further studies are desperately needed to identify the underlying causes in order to better understand and overcome the declining trend in survival among AYA bladder cancer patients.

#### 4.3.4. Acute Lymphatic Leukaemia

Paediatric cancer patients are often treated more aggressively and studies assessing the effect of paediatric-inspired regimens in ALL AYA patients have reported improved survival outcomes (more so in paediatric vs. adult centres) [8,59,60]. Impressive gains in AYA ALL patients have likewise been demonstrated following therapeutic advances [59]. In the Netherlands, a 25% increase in overall survival at five-years of follow-up among ALL patients aged 18–39 years was observed from 2001–2006 through 2007–2012, which was mainly explained by the implementation of more intensive paediatric-based chemotherapeutic regimens in 2005 [60,61]. Additionally suggested were the increased application of allogeneic stem cell therapy and the introduction of tyrosine kinase inhibitors since the early 2000s [60]. While survival outcomes at five-years of follow-up among AYAs with ALL still lack behind those of paediatric ALL patients (85–90% overall survival), relative survival outcomes have become encouragingly high around 70–75% in 2010–2016 [62].

#### 4.3.5. Chronic Myeloid Leukaemia

The introduction of (life-long) tyrosine kinase inhibitor treatment, which specifically targets BCR-ABL, often found in CML patients, likely also resulted in the high survival gains and favourable outcomes that were observed for all CML patients at AYA age in this study [9,13,63].

#### 4.3.6. Lung, Liver, Pancreas, and Stomach Carcinomas

Likely also related to therapeutic advances are the improvements in survival among AYAs with lung, liver, pancreas, and stomach carcinomas (more prominent in males) [64,65,66,67]. Nevertheless, survival outcomes for these cancers in AYAs have remained relatively poor (<60%), which is characteristic for these cancer types regardless of patient age [52,68,69,70,71,72]. Studies focusing on young patients (not necessarily AYAs), have shown that younger patients (aged < 50 years) with lung, liver, pancreas, and stomach carcinomas are more often diagnosed with more aggressive disease with higher grade, more advanced stage and higher metastatic rates when compared with older populations [64,65,68,69,70,71,72]. This likely relates in part to delays in diagnosis and higher frequencies of driver mutations (e.g., EGFR and ALK mutations in lung cancer patients) [72,73,74]. In addition, survival outcomes for younger pancreatic and lung cancer patients did not improve drastically when treated with more aggressive treatment regimens [67,75]. This might explain why younger patients, in some studies, show similar or worse overall survival outcomes compared with older populations even though they generally suffer from less comorbidities and are better able to resist more intensive treatment regimens [65,68,69,72,73,76]. Considering the more aggressive disease biology and that younger patients have more potential life-years to lose, these cancers should receive increased attention in future research, as literature on these cancers among AYAs is scarce [9,65,71,72,73]. Additionally, consideration of genetic testing might help improve survival outcomes for young lung cancer patients, as their higher mutation rates provide suitable targets for more personalised treatment strategies [52,72].

#### 4.3.7. Colorectal Carcinomas

Also warranting increased attention, is the limited improvement in the survival of AYA CRC patients (<9% improvement between 1990–1999 and 2010–2016), despite being treated more aggressively compared to older patients [77]. Delays in diagnosis (≥6 months on average in patients aged < 40 years) and lack of dedicated screening programmes are likely in part responsible for the higher stages of disease often found among AYAs and could provide some explanation for the lack in progress among AYA CRC patients in this study [43,78]. A growing body of evidence also suggests that CRCs among AYAs are biologically different from those in older adults, exhibiting a more aggressive phenotype exemplified by the increased prevalence of mucinous histology, signet ring cells, higher microsatellite instability, and mismatch-repair gene mutations [43,44,78]. Adoption of screening and novel treatment regimens incorporating the distinct tumour biology have the potential to substantially impact the survival of AYA CRC and should be explored [43].

#### 4.3.8. Soft Tissue and Bone Sarcomas

With the exception of Kaposi sarcoma, the survival of AYA sarcoma patients has remained relatively poor, especially for those with rhabdomyosarcomas, which despite its chemosensitivity has a high risk on local and distant recurrence and death [79]. It has been well established that the survival of sarcomas decreases considerably with age, as exemplified by recent data showing significantly worse survival outcomes for AYA compared to paediatric sarcoma patients in Europe between 2000–2007 [12]. However, AYA age was not found to be an independent risk factor for bone sarcoma-related death in a recent Japan study [80]. Outcome discrepancies between AYA and paediatric patients for histologically similar sarcomas have been attributed to differences in tumour biology, but also to differences in treatment (e.g., specific guidelines, adherence to protocols, and treatment dose intensity) and organisation of care (e.g., care centralisation in expert centres and wide collaborations on national and international levels) [79,80,81,82]. Compared to children, AYA sarcoma patients also tend to have a higher stage at diagnosis resulting from patient and doctor delay and incorrect diagnosis [79,81,83]. Low availability and enrolment of AYAs in clinical trials (5–34% in AYAs vs. 70–80% in children) have also been implicated [79,81,84]. Addressing these factors will likely improve clinical outcomes among AYAs and initiatives are already under way to achieve this [85].

### 4.4. Trends in Mortality

Promising declines in AYA cancer mortality with time have been observed since the 1970s [24,25,86]. Our analyses showed that observed mortality rates and declines over time were slightly higher among female AYAs, particularly among those aged 30 years and above. Although not directly stated, more prominent declines for a majority of specific cancer types among female AYAs were also observed by Close and colleagues [24]. A higher mortality burden in females (ages 20–39 years) was reported by Fidler et al. in 2012, and although not studied here, their investigation showed that breast and cervix/uterus cancers were among the major contributors of cancer-related deaths among females (25.1% and 14.3%, respectively) [15]. In contrast, a recent analyses with SEER data between 2008–2017 showed a similar 1% average decline per year for both sexes, whereas it levelled off for women aged 30–39 years, likely reflecting stabilizing breast cancer mortality trends [25]. Another recent American study (2020) on time-related long-term mortality trends among five-year AYA cancer survivors likewise showed no disparities between sexes at 5-, 10-, and 20-years of follow-up [86]. However, their analyses did show marked improvements in late all-cause mortality that was mainly driven by temporal decreases in the mortality of primary cancers, but time-related improvements were less consistent across cancer types [86]. As suggested by the authors, these findings likely reflect improvements in cancer therapies and refinement of treatment strategies and might help identify priority groups for long-term surveillance to reduce late mortality from cancer [86]. Continuing monitoring of (long-term) mortality trends among AYAs is therefore important.

### 4.5. Clinical Implications

AYAs have a distinct distribution of cancers and compared to earlier and older cancer patients, exhibit unique cancer trends (e.g., the distinct rise in CRC). Underlying these trends are several modifiable (e.g., obesity and participation in clinical trials) and non-modifiable factors (e.g., host age, tumour biology, and genetic predisposition). Although research efforts centred on AYA oncology have drastically increased in the past decade following the “Closing the gap” report [1], the AYA population is still underrepresented in cancer research and clear understanding of the underlying biological/genetic drivers for several cancer types is still lacking. Considering that the cancer burden is increasing in most countries worldwide and that AYAs have a large proportion of their expected lifespans remaining (thus contributing substantially to the economy), increased focus should be directed towards improving primary and secondary prevention of AYA cancers. To answer more complex research questions, large prospective datasets with detailed patient, clinical, and treatment information on AYA cancer are needed. Considering that such data resources are limited, increased efforts are needed to initiate such data collections [87]. In line with recent recommendations, increasing availability and participation in clinical trials by overcoming known barriers is also crucial to advance AYA oncology outcomes. Additionally, increased collaboration between experts from paediatric and the adult oncology at both the national and international level is needed to best address the distinct needs and challenges that this unique population faces [7,10].

### 4.6. Strenghts and Limitations

Findings in this study are based on almost three decades of high-quality data, systematically obtained by trained registrars from the population-based NCR. Use of this data can be considered a major strength as selection bias is not present due to the inclusion of the entire Dutch population regardless of age at the time of cancer diagnosis. Based on these data, we were able to provide a long-term comprehensive description on trends in incidence, survival, and mortality specific to the AYA population. Stratification in five-year age groups allowed for a detailed assessment of age-specific trends within the AYA population, which is another major strength of this study considering that AYAs are often grouped together, masking important age-related differences. Our study also has a number of limitations. First, the interpretation of results for rare cancers should be done with caution as random fluctuations due to low number of cases may erroneously appear as noteworthy trends. Second, low case numbers during certain sub-analyses lead to the inability for various cancer types to calculate average annual percentage change statistics. Finally, it could be that the trends observed in this study are the results from using the entire AYA age range and all stages combined, whereas some trends might only become apparent with more narrow age groups and individual disease stages.

## 5. Conclusions

As demonstrated in this study, over the past three decades there has been a considerable rise in cancer burden among AYAs in the Netherlands. Rising trends in incidence were found for most cancer types. The survival greatly improved and now well exceeds above 80% at five-years of follow-up for all cancers combined and regardless of sex. However, progress was limited and survival outcomes have remained relatively poor (<60%) for several distinct cancer types, which is of great concern given the worldwide rise in AYA cancer incidence. Cancer mortality trends steadily declined for most AYAs since 1990, likely due to more effective treatment options. Increased knowledge of AYA cancers and their distinct biology is needed to better explain the unique cancer trends observed within the AYA population and to better understand the outcome disparities observed between males and females. As such, there is a need for more detailed studies based on comprehensive data sources that examine individual cancer types by age, sex, and treatment patterns in AYAs with cancer. Increased focus should also be directed towards improving AYA cancer awareness among clinicians and patients, and the inclusion of AYAs with cancer in clinical trials to allow for better care optimisation.

## Figures and Tables

**Table 1:** Total and average number of new cancer cases per year, incidence rates and average annual percentage change (AAPC) estimates of all adolescents and young adults (AYAs) aged 15–39 years at time of diagnosis in the Netherlands between 1990–2016.

Population Characteristics	Total Number of Cases	Average Number of New Cases per Year				Age-Standardised Incidence Rates (ESR) per 100,000 Person-Year ^††^				AAPC (95% CI) §§
		1990–2016	1990–1999	2000–2009	2010–2016	1990–2016	1990–1999	2000–2009	2010–2016	
All cancers	95,228	3527	3339	3641	3634	61.2	54.6	62.6	70.3	1.37 * (1.24, 1.51)
Sex										
Male	39,038	1446	1348	1487	1526	50.0	43.6	51.7	58.8	1.23 * (0.83, 1.64)
Female	56,190	2081	1991	2153	2108	72.6	66.1	73.7	81.8	1.18 * (1.03, 1.34)
Age (years)										
15–19	5434	201	182	204	225	20.6	19.1	20.9	22.3	0.88 * (0.58, 1.18)
20–24	8974	332	312	326	370	31.6	27.6	33.5	35.1	1.24 * (0.83, 1.66)
25–29	15,421	571	554	548	629	50.8	43.1	53.3	60.8	1.91 * (1.65, 2.17)
30–34	25,601	948	921	963	966	80.7	71.3	81.9	95.5	1.60 * (1.41, 1.79)
35–39	39,798	1474	1369	1599	1445	122.3	112.0	123.3	137.7	1.01 * (0.67, 1.35)
Cancer types *										
1. Leukaemia’s	3556	132	133	132	129	2.4	2.7	2.6	2.6	0.61 * (0.12, 1.10)
1.1 Acute lymphoid leukaemia’s	1068	40	40	39	40	0.8	0.7	0.8	0.8	0.62 (−0.14, 1.39)
1.2 Acute myeloid leukaemia’s	1502	56	62	53	50	1.0	1.0	1.0	1.0	−0.24 (−0.91, 0.43)
1.3 Chronic myeloid leukaemia’s	632	23	21	25	24	0.4	0.4	0.5	0.5	1.99 * (0.49, 3.51)
1.4 Other and unspecified leukaemia’s	354	13	11	14	14	0.2	0.2	0.2	0.3	1.51 (−0.22, 3.27)
2. Lymphomas	10,211	378	368	384	385	6.8	7.4	7.7	7.7	1.06 * (0.84, 1.27)
2.1 Non-Hodgkin lymphomas	4740	176	175	180	171	3.1	2.9	3.1	3.3	0.79 * (0.38, 1.19)
2.2 Hodgkin lymphomas	5471	203	194	204	214	3.7	3.3	3.8	4.1	1.27 * (0.87, 1.67)
3. CNS and other intracranial and intraspinal neoplasm	4644	172	178	171	165	3.1	3.6	3.4	3.3	0.41 (−0.02, 0.85)
3.1 Astrocytoma’s †	2842	105	111	103	101	1.9	1.9	1.8	2.0	0.31 (−0.26, 0.89)
3.2 Other gliomas	938	35	36	37	31	0.6	0.6	0.6	0.6	−1.02 (−3.10, 1.10)
3.3 Ependymomas	299	11	11	11	11	0.2	0.2	0.2	0.2	−0.23 (−1.90, 1.46)
3.4 Medulloblastomas and other PNET §	219	8	8	9	7	0.2	0.1	0.2	0.1	0.17 (−1.95, 2.34)
3.5 Other specified intracranial and intraspinal neoplasms	81	3	3	2	3	0.1	0.1	0.0	0.1	NA
3.6 Unspecified intracranial and intraspinal neoplasms ‡	265	10	9	9	12	0.2	0.1	0.2	0.2	2.80 * (1.17, 4.46)
4. Osseous and Chondromatous neoplasms	1434	53	52	54	55	1.0	1.0	1.1	1.1	0.79 * (0.23, 1.36)
4.1 Osteosarcomas	547	20	21	19	20	0.4	0.4	0.4	0.4	−0.04 (−1.06, 1.00)
4.2 Chondrosarcomas	256	9	9	9	11	0.2	0.2	0.2	0.2	1.86 * (0.47, 3.37)
4.3 Ewing tumours	453	17	16	18	17	0.3	0.3	0.4	0.3	1.04 * (0.02, 2.06)
4.4 Other specified and unspecified bone tumours	178	7	6	7	6	0.1	0.1	0.1	0.1	0.85 (−1.13, 2.86)
5. Soft tissue sarcomas	3803	141	171	134	107	2.5	3.4	2.7	2.1	−2.05 * (−2.96, −1.14)
5.1 Fibromatous neoplasms	1057	39	46	38	31	0.7	0.8	0.7	0.6	−1.24 * (−1.91, −0.57)
5.2 Rhabdomyosarcomas	202	7	8	7	7	0.1	0.2	0.1	0.2	−0.43 (−1.91, 1.08)
5.3.1.1 Specified soft tissue sarcomas (excluding Kaposi sarcoma)	1599	59	62	63	50	1.0	1.0	1.1	1.0	−0.40 (−1.11, 0.31)
5.3.1.2 Kaposi sarcomas	690	26	45	17	10	0.4	0.7	0.3	0.2	−8.56 * (−12.36, −4.61)
5.3.2 Unspecified soft tissue sarcomas	255	9	9	10	8	0.2	0.2	0.2	0.2	0.48 (−1.12, 2.10)
6. Germ cell and Trophoblastic neoplasms	11,620	430	333	455	534	7.8	6.7	9.1	10.7	3.41 * (2.88, 3.94)
6.1 Germ cell and trophoblastic neoplasms of the gonads	11,076	410	312	435	515	7.3	5.2	7.9	10.0	3.57 * (3.03, 4.12)
6.2 Germ cell and trophoblastic neoplasms of non-gonadal sites	544	20	21	21	19	0.4	0.4	0.4	0.4	0.24 (−1.00, 1.50)
7. Melanoma and skin carcinomas	16,969	628	540	692	665	11.3	10.8	13.8	13.3	1.95 * (1.45, 2.44)
7.1 Melanoma	15,987	592	508	655	622	10.2	8.3	11.3	12.0	1.92 * (1.44, 2.40)
7.2 Skin carcinomas	982	36	32	37	43	0.6	0.5	0.6	0.8	2.76 * (1.69, 3.84)
8. Carcinomas	41,703	1545	1514	1574	1547	27.8	30.3	31.5	30.9	0.90 * (0.63, 1.17)
8.1 Thyroid carcinomas	3298	122	97	124	154	2.1	1.6	2.2	3.0	3.36 * (2.88, 3.85)
8.2 Head and neck carcinomas #	1831	68	68	73	61	1.2	1.1	1.2	1.2	0.13 (−0.68, 0.94)
8.3 Carcinomas of trachea, bronchus, and lung	2144	79	91	79	65	1.3	1.5	1.3	1.3	−0.86 * (−1.24, −0.46)
8.4 Carcinomas of breast	18,165	673	628	708	687	11.2	10.2	11.4	13.3	1.27 * (0.75, 1.78)
8.5.1 Carcinomas of kidney	982	36	31	38	42	0.6	0.5	0.6	0.8	2.95 * (1.89, 4.01)
8.5.2 Carcinomas of bladder	439	16	17	17	15	0.3	0.3	0.3	0.3	0.13 (−1.07, 1.35)
8.5.3 Carcinomas of gonads	1279	47	64	39	36	0.8	1.0	0.7	0.7	−2.27 * (−3.81, −0.71)
8.5.4 Carcinomas of cervix and uterus	6088	225	247	208	220	3.8	4.0	3.4	4.3	0.34 (−0.37, 1.05)
8.6.1 Carcinomas of colon/rectum	3629	134	118	145	142	2.3	1.9	2.4	2.8	1.85 * (1.34, 2.37)
8.6.2 Carcinomas of the stomach	939	35	39	34	30	0.6	0.6	0.6	0.6	−0.35 (−1.33, 0.64)
8.6.3 Carcinomas of liver and intrahepatic bile ducts	259	10	9	9	11	0.2	0.2	0.2	0.2	2.08 * (0.64, 3.54)
8.6.4 Carcinomas of pancreas	432	16	14	17	17	0.3	0.2	0.3	0.3	1.72 * (0.22, 3.24)
8. Carcinomas of other ill-defined sites ¶	2218	82	93	82	67	1.4	1.5	1.4	1.3	−0.86 * (−1.44, −0.27)
9. Miscellaneous specified neoplasms, NOS **	1057	39	40	38	41	0.7	0.8	0.8	0.8	0.93 * (0.06, 1.80)
10. Unspecified malignant neoplasms	231	9	10	8	7	0.2	0.2	0.2	0.1	−0.95 (−3.07, 1.20)

Abbreviations: ESR = European Standard Rates, CI = Confidence Interval, CNS = Central Nervous System, PNET = Primitive Neuro-Ectodermal Tumours, ICD-O = the International Classification of Diseases for Oncology. * Cancers in the Netherlands Cancer Registry are coded using the ICD-O valid at the time of diagnosis; 1st edition before 1993, 2nd edition between 1993 and 2000 and 3th edition since 2001. Cancer types were grouped based on the Surveillance, Epidemiology, and End Results Program (SEER) AYA site recode adapted classification scheme. **†** Including specified low-grade astrocytic tumours, glioblastomas and anaplastic astrocytoma, and astrocytoma, NOS. **§** Including medulloblastomas and supratentorial PNET. **‡** Including unspecified malignant intracranial and intraspinal neoplasms and unspecified benign/border intracranial and intraspinal neoplasms. **#** Including carcinomas of the nasopharynx, other sites in the lip, oral cavity and pharynx, and nasal cavity, mid ear, sinuses, larynx and other ill-defined head and neck tumours. ¶ Including carcinomas of other ill-defined sites of the genitourinary tract, gastrointestinal tract, NOS, and adrenocortical carcinomas. ** Including Wilms tumours, neuroblastoma, other paediatric and embryonal tumours, NOS, paraganglioma and glomus tumours, other specified gonadal tumours, myeloma, mast cell, misc lymphoreticular neoplasms, NOS and other specified neoplasms, NOS. **††** Incidence rates were calculated per 100,000 person-years using the mid-year population size as person-time denominator and standardized with weights from the 1976 European Standard Population. **§§** AAPC and *p*-value outcomes denoted with “NA” could not be computed due to having zero counts in one or more individual years of diagnosis. * Indicates significant trends (*p* < 0.05).

**Table 2:** Number at risk and five-year relative survival estimates with 95% confidence intervals of adolescent and young adult (AYA) cancer patients aged 15–39 years at time of diagnosis in the Netherlands between 1990–2016 and presented by sex, age, cancer type and period of diagnosis.

Population Characteristics	Period of Diagnosis								*p*-Value ††
	1990–2016		1990–1999		2000–2009		2010–2016		
	*n* at Risk	% RS (95% CI)	*n* at Risk	% RS (95% CI)	*n* at Risk	% RS (95% CI)	*n* at Risk	% RS (95% CI)	
All cancers	95,228	79.9 (79.6, 80.2)	33,386	73.7 (73.2, 74.1)	36,405	81.3 (80.9, 81.7)	25,437	86.4 (86.0, 86.9)	0.00
Sex									
Male	39,038	78.1 (77.7, 78.5)	13,481	70.0 (69.2, 70.8)	14,873	80.2 (79.5, 80.8)	10,684	85.7 (85.0, 86.4)	0.00
Female	56,190	81.2 (80.8, 81.5)	19,905	76.2 (75.6, 76.8)	21,532	82.2 (81.6, 82.7)	14,753	86.9 (86.3, 87.5)	0.00
Age (years)									
15–19	5434	80.5 (79.4, 81.5)	1824	73.7 (71.6, 75.6)	2038	81.9 (80.2, 83.5)	1572	86.7 (84.8, 88.3)	0.00
20–24	8974	84.1 (83.3, 84.9)	3123	77.9 (76.4, 79.4)	3264	85.6 (84.3, 86.8)	2587	89.9 (88.6, 91.1)	0.00
25–29	15,421	83.2 (82.6, 83.8)	5540	77.9 (76.7, 78.9)	5477	84.1 (83.1, 85.1)	4404	89.2 (88.2, 90.2)	0.00
30–34	25,601	81.0 (80.5, 81.5)	9209	74.8 (73.9, 75.7)	9632	82.7 (82.0, 83.5)	6760	87.2 (86.4, 88.1)	0.00
35–39	39,790	76.9 (76.5, 77.3)	13,690	70.2 (69.5, 71.0)	15,994	78.6 (78.0, 79.2)	10,114	83.7 (82.9, 84.5)	0.00
Cancer types *									
1. Leukaemia’s	3556	59.2 (57.5, 60.8)	1334	47.1 (44.3, 49.7)	1321	62.3 (59.6, 64.9)	901	72.9 (69.8, 75.8)	0.00
1.1 Acute lymphoid leukaemia’s	1068	56.2 (53.1, 59.2)	396	43.5 (38.5, 48.4)	391	58.4 (53.3, 63.1)	281	71.8 (65.9, 76.9)	0.00
1.2 Acute myeloid leukaemia’s	1502	48.9 (46.3, 51.4)	616	39.9 (36.0, 43.7)	533	50.6 (46.3, 54.8)	353	62.3 (56.8, 67.3)	0.00
1.3 Chronic myeloid leukaemia’s	632	78.5 (75.1, 81.6)	211	57.5 (50.5, 63.9)	254	86.9 (82.0, 90.5)	167	92.9 (87.3, 96.2)	0.00
1.4 Other and unspecified leukaemia’s	354	77.1 (72.2, 81.2)	111	80.2 (71.3, 86.7)	143	72.8 (64.7, 79.5)	100	80.0 (70.2, 86.9)	0.95
2. Lymphomas	10,211	85.7 (85.0, 86.4)	3680	78.7 (77.4, 80.0)	3837	87.5 (86.4, 88.5)	2694	93.0 (91.9, 94.0)	0.00
2.1 Non-Hodgkin lymphomas	4740	76.1 (74.9, 77.3)	1745	65.0 (62.7, 67.3)	1797	79.5 (77.5, 81.3)	1198	87.6 (85.5, 89.4)	0.00
2.2 Hodgkin lymphomas	5471	94.0 (93.3, 94.7)	1935	91.1 (89.7, 92.3)	2040	94.6 (93.5, 95.5)	1496	97.4 (96.3, 98.2)	0.00
3. CNS and other intracranial and intraspinal neoplasm	4644	58.8 (57.3, 60.2)	1780	54.3 (51.9, 56.6)	1707	57.7 (55.3, 60.0)	1157	67.8 (64.8, 70.6)	0.00
3.1 Astrocytoma’s †	2842	51.5 (49.7, 53.4)	1110	48.0 (45.1, 51.0)	1027	49.6 (46.4, 52.6)	705	60.3 (56.3, 64.1)	0.00
3.2 Other gliomas	938	68.1 (65.0, 71.1)	355	63.4 (58.1, 68.2)	367	67.2 (62.1, 71.8)	216	78.7 (72.3, 83.9)	0.00
3.3 Ependymomas	299	84.1 (79.3, 87.9)	114	80.9 (72.3, 87.1)	110	86.6 (78.6, 91.8)	75	84.7 (73.0, 91.7)	0.27
3.4 Medulloblastomas and other PNET §	219	64.1 (57.2, 70.2)	82	65.4 (53.9, 74.7)	86	59.4 (48.3, 69.0)	51	69.3 (52.9, 80.9)	0.83
3.5 Other specified intracranial and intraspinal neoplasms	81	61.6 (49.9, 71.3)	34	62.0 (43.6, 76.0)	24	58.5 (36.5, 75.2)	23	62.7 (38.4, 79.7)	0.77
3.6 Unspecified intracranial and intraspinal neoplasms ‡	265	69.4 (63.3, 74.7)	85	48.7 (37.6, 58.9)	93	73.3 (63.0, 81.2)	87	85.9 (75.4, 92.1)	0.00
4. Osseous and Chondromatous neoplasms	1434	64.1 (61.5, 66.5)	515	57.5 (53.1, 61.7)	535	63.9 (59.7, 67.9)	384	73.1 (67.9, 77.6)	0.00
4.1 Osteosarcomas	547	61.2 (56.9, 65.2)	211	56.4 (49.4, 62.8)	194	60.8 (53.5, 67.3)	142	69.3 (60.3, 76.8)	0.02
4.2 Chondrosarcomas	256	82.2 (76.8, 86.5)	88	75.2 (64.7, 83.0)	90	82.3 (72.6, 88.9)	78	90.6 (81.0, 95.6)	0.01
4.3 Ewing tumours	453	48.7 (44.0, 53.3)	156	38.9 (31.2, 46.5)	178	48.7 (41.2, 55.9)	119	60.5 (50.2, 69.3)	0.00
4.4 Other specified and unspecified bone tumours	178	85.9 (79.7, 90.3)	60	83.6 (71.5, 91.0)	73	86.5 (76.3, 92.6)	45	88.2 (73.5, 95.1)	0.57
5. Soft tissue sarcomas	3803	69.3 (67.8, 70.8)	1709	60.3 (57.9, 62.6)	1344	76.3 (73.9, 78.5)	750	77.7 (74.3, 80.7)	0.00
5.1 Fibromatous neoplasms	1057	94.9 (93.3, 96.1)	460	91.6 (88.6, 93.9)	378	96.8 (94.3, 98.3)	219	98.8 (96.0, 99.8)	0.00
5.2 Rhabdomyosarcomas	202	40.6 (33.7, 47.4)	84	35.8 (25.7, 46.0)	66	42.5 (30.5, 54.0)	52	45.3 (30.6, 58.9)	0.17
5.3.1.1 Specified soft tissue sarcomas (excluding Kaposi sarcoma)	1599	67.8 (65.4, 70.1)	619	65.3 (61.4, 69.0)	630	69.0 (65.2, 72.5)	350	69.6 (64.2, 74.5)	0.11
5.3.1.2 Kaposi sarcomas	690	50.3 (46.4, 54.0)	452	29.8 (25.6, 34.1)	168	87.8 (81.7, 92.0)	70	96.0 (87.4, 98.9)	0.00
5.3.2 Unspecified soft tissue sarcomas	255	47.3 (40.9, 53.3)	94	41.1 (31.1, 50.9)	102	48.5 (38.5, 57.9)	59	54.6 (40.5, 66.7)	0.11
6. Germ cell and Trophoblastic neoplasms	11,620	96.9 (96.5, 97.2)	3331	95.1 (94.3, 95.8)	4551	96.8 (96.3, 97.3)	3738	98.5 (98.0, 98.9)	0.00
6.1 Germ cell and trophoblastic neoplasms of the gonads	11,076	97.6 (97.3, 97.9)	3123	96.3 (95.5, 96.9)	4345	97.6 (97.1, 98.1)	3608	98.8 (98.3, 99.2)	0.00
6.2 Germ cell and trophoblastic neoplasms of non-gonadal sites	544	81.7 (78.2, 84.8)	208	77.2 (70.8, 82.4)	206	80.3 (74.1, 85.1)	130	91.1 (84.2, 95.1)	0.00
7. Melanoma and skin carcinomas	16,969	92.5 (92.1, 92.9)	5400	90.0 (89.1, 90.7)	6916	92.9 (92.3, 93.5)	4653	95.1 (94.4, 95.8)	0.00
7.1 Melanoma	15,987	92.3 (91.9, 92.7)	5084	89.7 (88.8, 90.5)	6550	92.7 (92.0, 93.3)	4353	95.1 (94.3, 95.7)	0.00
7.2 Skin carcinomas	982	95.4 (93.8, 96.6)	316	93.7 (90.3, 96.0)	366	96.5 (93.9, 98.1)	300	96.0 (92.8, 97.8)	0.18
8. Carcinomas	41,703	74.8 (74.3, 75.2)	15,139	69.1 (68.4, 69.9)	15,735	75.9 (75.3, 76.6)	10,829	81.4 (80.6, 82.2)	0.00
8.1 Thyroid carcinomas	3298	98.3 (97.7, 98.7)	974	98.1 (96.9, 98.8)	1244	98.4 (97.5, 99.0)	1080	98.3 (97.2, 99.0)	0.69
8.2 Head and neck carcinomas #	1831	79.4 (77.4, 81.2)	680	73.7 (70.1, 76.8)	727	81.6 (78.5, 84.3)	424	85.1 (81.1, 88.3)	0.00
8.3 Carcinomas of trachea, bronchus, and lung	2144	29.1 (27.2, 31.1)	905	24.2 (21.5, 27.1)	785	29.5 (26.3, 32.7)	454	37.9 (33.2, 42.6)	0.00
8.4 Carcinomas of breast	18,165	82.6 (82.1, 83.2)	6275	76.0 (74.9, 77.0)	7083	84.7 (83.8, 85.5)	4807	89.4 (88.4, 90.3)	0.00
8.5.1 Carcinomas of kidney	982	79.8 (77.1, 82.2)	305	71.8 (66.3, 76.5)	382	81.4 (77.0, 85.0)	295	86.5 (81.9, 90.1)	0.00
8.5.2 Carcinomas of bladder	439	58.0 (53.2, 62.6)	169	64.4 (56.6, 71.2)	168	53.8 (45.9, 61.0)	102	54.1 (43.5, 63.6)	0.04
8.5.3 Carcinomas of gonads	1279	66.6 (63.9, 69.1)	635	69.9 (66.1, 73.3)	392	62.9 (57.9, 67.5)	252	62.5 (55.5, 68.7)	0.07
8.5.4 Carcinomas of cervix and uterus	6088	87.2 (86.3, 88.0)	2466	86.3 (84.9, 87.7)	2079	86.7 (85.1, 88.1)	1543	89.3 (87.5, 90.9)	0.01
8.6.1 Carcinomas of colon/rectum	3629	64.1 (62.5, 65.7)	1182	59.2 (56.3, 61.9)	1450	65.8 (63.2, 68.2)	997	67.7 (64.5, 70.7)	0.00
8.6.2 Carcinomas of the stomach	939	27.4 (24.6, 30.4)	385	21.8 (17.8, 26.0)	343	30.6 (25.7, 35.6)	211	33.7 (27.2, 40.3)	0.00
8.6.3 Carcinomas of liver and intrahepatic bile ducts	259	26.6 (21.1, 32.3)	89	17.2 (10.0, 26.0)	94	24.3 (16.1, 33.4)	76	40.0 (28.0, 51.8)	0.00
8.6.4 Carcinomas of pancreas	432	29.4 (25.2, 33.8)	144	23.0 (16.5, 30.2)	166	21.4 (15.5, 27.9)	122	47.8 (38.5, 56.4)	0.00
8. Carcinomas of other ill-defined sites ¶	2218	39.2 (37.2, 41.3)	930	31.4 (28.5, 34.5)	822	40.2 (36.8, 43.6)	466	53.1 (48.3, 57.7)	0.00
9. Miscellaneous specified neoplasms, NOS **	1057	71.5 (68.6, 74.2)	397	66.2 (61.3, 70.7)	375	71.9 (67.1, 76.2)	285	79.0 (73.6, 83.5)	0.00
10. Unspecified malignant neoplasms	231	32.2 (26.2, 38.3)	101	20.9 (13.6, 29.3)	84	35.5 (25.3, 45.7)	46	51.2 (35.7, 64.8)	0.00

Abbreviations: RS = Relative Survival, CI = Confidence Intervals, CNS= Central Nervous System, PNET = Primitive Neuro-Ectodermal Tumours, ICD-O = the International Classification of Diseases for Oncology. * Cancers in the Netherlands Cancer Registry are coded using the ICD-O valid at the time of diagnosis; 1st edition before 1993, 2nd edition between 1993 and 2000 and 3th edition since 2001. Cancer types were grouped based on the Surveillance, Epidemiology, and End Results Program (SEER) AYA site recode adapted classification scheme. † Including specified low-grade astrocytic tumours, glioblastomas and anaplastic astrocytoma, and astrocytoma, NOS. § Including medulloblastomas and supratentorial PNET. ‡ Including unspecified malignant intracranial and intraspinal neoplasms and unspecified benign/border intracranial and intraspinal neoplasms. ^#^ Including carcinomas of the nasopharynx, other sites in the lip, oral cavity and pharynx, and nasal cavity, mid ear, sinuses, larynx and other ill-defined head and neck tumours. ¶ Including carcinomas of other ill-defined sites of the genitourinary tract, gastrointestinal tract, NOS, and adrenocortical carcinomas. ** Including Wilms tumours, neuroblastoma, other paediatric and embryonal tumours, NOS, paraganglioma and glomus tumours, other specified gonadal tumours, myeloma, mast cell, misc lymphoreticular neoplasms, NOS and other specified neoplasms, NOS. ^††^ *p*-values were obtained from a likelihood ratio test comparing a generalized linear model including the midpoint of the calendar period and a model without calendar period. All models assumed a Poisson distribution for the observed number of deaths. Significance was determined if *p* < 0.05.

## Supplementary Materials

The following are available online at https://www.mdpi.com/2072-6694/12/11/3421/s1, Table S1. Total and average number of new cancer cases per year, incidence rates and AAPCs in male AYAs aged 15–39 years at time of diagnosis in the Netherlands between 1990–2016, Table S2. Total and average number of new cancer cases per year, incidence rates and AAPCs in female AYAs aged 15–39 years at time of diagnosis in the Netherlands between 1990–2016, Table S3. Number at risk and five-year relative survival estimates with 95% confidence intervals of male AYA cancer patients aged 15–39 years at time of diagnosis in the Netherlands by age, cancer type and period of diagnosis between 1990–2016, Table S4. Number at risk and five-year relative survival estimates with 95% confidence intervals of female AYA cancer patients aged 15–39 years at time of diagnosis in the Netherlands by sex, age, cancer type, and period of diagnosis between 1990–2016, Table S5. Absolute numbers of cancer-specific mortality (ICD-10 code C00-C97) cases among adolescents and young adults (AYAs) in the Netherlands between 1990–2016.
