# Supplementary material for: Incidence, Survival, and Mortality Trends of Cancers Diagnosed in Adolescents and Young Adults (15–39 Years): A Population-Based Study in The Netherlands 1990–2016
Source: Cancers (Basel). 2020 Nov 18;12(11):3421. doi: 10.3390/cancers12113421 (PMC7698904; doi:10.3390/cancers12113421)
Supplement: Supplementary file 1 [file cancers-12-03421-s001.pdf]

cancers

MDPI

Supplementary Materials:

Incidence, Survival and Mortality Trends of Cancers Diagnosed in Adolescents and Young Adults (15–39 Years): A Population-Based Study in the Netherlands 1990–2016

Daniël J. van der Meer, Henrike E. Karim-Kos, Marianne van der Mark, Katja K.H. Aben, Rhodé M. Bijlsma, Anita W. Rijneveld, Winette T.A. van der Graaf and Olga Husson

Table S1. Total and average number of new cancer cases per year, incidence rates and average annual percentage change (AAPC) estimates of male adolescents and young adults (AYAs) aged 15–39 years at time of diagnosis in the Netherlands between 1990–2016.

Population Characteristics	Total Number of Cases	Average Number of New Cases per Year				Age-Standardised Incidence Rates (ESR) per 100,000 Person-Year††				AAPC (95% CI)§§
Period of Diagnosis		1990–2016	1990–1999	2000–2009	2010–2016	1990–2016	1990–1999	2000–2009	2010–2016	
All cancers	39,038	1,446	1,348	1,487	1,526	50.0	43.6	51.7	58.8	1.23* (0.83, 1.64)
Age (years)										
15–19	3,066	114	101	117	126	22.7	20.7	23.5	24.5	0.98* (0.63, 1.33)
20–24	4,961	184	170	181	207	34.5	29.6	36.8	38.9	1.42* (0.95, 1.88)
25–29	7,474	277	265	268	306	48.6	40.4	51.9	58.7	2.09* (1.65, 2.52)
30–34	9,987	370	351	374	390	62.1	53.3	63.1	77.0	1.38* (0.65, 2.13)
35–39	13,550	502	460	547	497	82.3	74.0	83.2	94.8	1.10* (0.62, 1.58)
All cancers, excluding gonadal germ cell tumours	27,940	1,035	1,032	1,052	1,014	35.7	33.4	36.1	39.1	0.36 (−0.02, 0.74)
Age (years)										
15–19	2,398	89	80	90	99	18.5	16.4	18.0	19.3	0.88* (0.42, 1.35)
20–24	3,051	113	115	107	119	21.8	20.0	21.7	22.3	0.49* (0.04, 0.94)
25–29	4,560	169	182	154	171	30.4	27.7	29.8	32.9	0.94* (0.50, 1.38)
30–34	6,860	254	260	254	246	42.9	39.4	42.8	48.6	1.03* (0.62, 1.45)
35–39	11,071	410	395	447	378	69.2	63.6	68.0	72.2	0.02 (−0.68, 0.72)
All cancers, excluding thyroid carcinomas	38,231	1416	1322	1458	1490	49.0	42.7	50.7	57.4	1.22* (0.81, 1.62)
Age (years)										
15–19	3,004	111	99	115	123	23.1	20.3	23.1	23.9	0.97* (0.61, 1.33)
20–24	4,873	180	167	178	203	34.8	29.0	36.2	38.1	1.42* (0.94, 1.91)
25–29	7,299	270	259	262	298	48.7	39.5	50.8	57.2	2.08* (1.65, 2.50)

30–34	9,778	362	345	367	380	61.1	52.3	61.9	74.9	1.34* (0.66, 2.02)
35–39	13,277	492	452	535	486	83.0	72.7	81.4	92.8	1.07* (0.58, 1.55)
All cancers, excluding KS and NHL	35,403	1,311	1,194	1,360	1,410	45.4	38.6	47.3	54.3	1.68* (1.33, 2.03)
Age (years)										
15–19	2,789	103	92	108	114	21.5	18.7	21.5	22.2	0.98* (0.59, 1.36)
20–24	4,590	170	157	168	192	32.8	27.3	34.2	36.0	1.47* (0.97, 1.97)
25–29	6,862	254	238	248	287	45.8	36.2	48.0	55.0	2.33* (1.85, 2.81)
30–34	8,984	333	304	343	360	56.2	46.0	57.8	71.0	2.10* (1.48, 2.74)
35–39	12,178	451	404	494	457	76.1	64.9	75.1	87.3	1.30* (0.68, 1.93)
Cancer types*										
1. Leukaemia's	2,028	75	76	73	77	2.7	2.5	2.6	2.7	0.67* (0.08, 1.27)
1.1 Acute lymphoid leukaemia's	664	25	24	24	26	0.9	0.9	0.9	1.0	0.66 (−0.19, 1.52)
1.2 Acute myeloid leukaemia's	732	27	31	25	26	1.0	1.0	0.9	1.0	−0.46 (−1.37, 0.46)
1.3 Chronic myeloid leukaemia's	385	14	13	15	15	0.5	0.4	0.5	0.6	2.24* (0.52, 4.00)
1.4 Other and unspecified leukaemia's	247	9	8	10	10	0.3	0.3	0.3	0.4	1.19 (−0.79, 3.20)
2. Lymphomas	5,960	221	218	226	218	7.7	7.0	7.9	7.6	0.86* (0.57, 1.15)
2.1 Non-Hodgkin lymphomas	2,994	111	111	113	107	3.9	3.6	3.9	4.1	0.67* (0.15, 1.19)
2.2 Hodgkin lymphomas	2,966	110	106	112	111	4.0	3.5	4.1	4.3	1.06* (0.59, 1.53)
3. CNS and other intracranial and intraspinal neoplasm	2,740	101	106	99	99	3.4	3.4	3.4	3.5	0.55 (−0.04, 1.15)
3.1 Astrocytoma's†	1,696	63	66	60	62	2.2	2.2	2.1	2.4	0.61 (−0.11, 1.33)
3.2 Other gliomas	548	20	21	22	18	0.7	0.7	0.7	0.7	−1.98 (−5.78, 1.97)
3.3 Ependymomas	171	6	7	6	6	0.2	0.2	0.2	0.2	−0.60 (−2.53, 1.36)
3.4 Medulloblastomas and other PNET§	135	5	5	5	5	0.2	0.2	0.2	0.2	NA
3.5 Other specified intracranial and intraspinal neoplasms	41	2	2	1	2	0.1	0.1	0.0	0.1	NA
3.6 Unspecified intracranial and intraspinal neoplasms‡	149	6	5	5	7	0.2	0.2	0.2	0.3	2.58* (0.81, 4.38)
4. Osseous and Chondromatous neoplasms	832	31	31	31	31	1.3	1.1	1.1	1.1	0.64 (−0.25, 1.54)
4.1 Osteosarcomas	332	12	13	11	12	0.5	0.5	0.4	0.5	0.02 (−1.39, 1.46)
4.2 Chondrosarcomas	140	5	5	5	6	0.2	0.2	0.2	0.2	1.50 (−0.44, 3.48)
4.3 Ewing tumours	275	10	10	11	10	0.4	0.3	0.4	0.4	0.80 (−0.82, 2.44)
4.4 Other specified and unspecified bone tumours	85	3	3	4	3	0.1	0.1	0.1	0.1	NA
5. Soft tissue sarcomas	2,214	82	107	75	57	2.7	3.4	2.5	2.0	−2.98* (−4.19, −1.75)
5.1 Fibromatous neoplasms	504	19	24	19	11	0.7	0.8	0.7	0.4	−2.74* (−4.04, −1.43)
5.2 Rhabdomyosarcomas	125	5	5	4	5	0.2	0.2	0.2	0.2	−0.14 (−2.08, 1.83)

5.3.1.1 Specified soft tissue sarcomas (excluding Kaposi sarcoma)	817	30	31	32	28	1.1	1.0	1.1	1.1	0.17 (−0.66, 1.00)
5.3.1.2 Kaposi sarcomas	641	24	43	14	9	0.8	1.4	0.5	0.4	−8.42* (−14.01, −2.46)
5.3.2 Unspecified soft tissue sarcomas	127	5	4	6	4	0.2	0.1	0.2	0.2	1.32 (−1.41, 4.13)
6. Germ cell and Trophoblastic neoplasms	11,098	411	316	436	512	13.7	9.8	15.5	17.9	3.60* (3.05, 4.14)
6.1 Germ cell and trophoblastic neoplasms of the gonads	10,755	398	302	422	502	14.2	9.7	15.1	19.3	3.73* (3.16, 4.31)
6.2 Germ cell and trophoblastic neoplasms of non-gonadal sites	343	13	14	14	10	0.5	0.5	0.5	0.4	−0.53 (−2.12, 1.08)
7. Melanoma and skin carcinomas	6,087	225	199	242	241	6.8	6.2	7.9	8.4	2.34* (0.30, 4.42)
7.1 Melanoma	5,630	209	184	224	221	7.3	5.9	7.6	8.5	2.30* (0.60, 4.02)
7.2 Skin carcinomas	457	17	14	18	20	0.6	0.5	0.6	0.8	2.60* (1.23, 3.99)
8. Carcinomas	7,457	276	276	284	266	8.0	8.8	9.0	9.3	−0.39 (−1.39, 0.61)
8.1 Thyroid carcinomas	807	30	26	29	36	1.1	0.8	1.0	1.4	2.56* (1.47, 3.65)
8.2 Head and neck carcinomas#	1,077	40	41	43	34	1.4	1.3	1.4	1.3	−0.20 (−1.11, 0.72)
8.3 Carcinomas of trachea, bronchus, and lung	963	36	39	36	31	1.2	1.2	1.2	1.2	−0.60 (−1.43, 0.25)
8.4 Carcinomas of breast	37	1	1	2	1	0.1	0.0	0.1	0.0	NA
8.5.1 Carcinomas of kidney	605	22	18	25	25	0.8	0.6	0.8	1.0	2.88* (1.64, 4.13)
8.5.2 Carcinomas of bladder	255	9	11	9	8	0.3	0.3	0.3	0.3	−0.41 (−1.82, 1.03)
8.5.3 Carcinomas of gonads	8	0	0	1	0	0.0	0.0	0.0	0.0	NA
8.5.4 Carcinomas of cervix and uterus										
8.6.1 Carcinomas of colon/rectum	1,823	68	60	71	73	2.3	1.9	2.3	2.8	1.88* (1.11, 2.66)
8.6.2 Carcinomas of the stomach	516	19	22	18	16	0.7	0.7	0.6	0.6	−0.93 (−2.33, 0.50)
8.6.3 Carcinomas of liver and intrahepatic bile ducts	150	6	5	5	6	0.2	0.2	0.2	0.2	1.78 (−0.06, 3.65)
8.6.4 Carcinomas of pancreas	199	7	7	8	7	0.3	0.2	0.3	0.3	0.79 (−1.15, 2.77)
8. Carcinomas of other ill-defined sites¶	1,017	38	45	37	29	1.3	1.4	1.2	1.1	−4.06* (−7.88, −0.07)
9. Miscellaneous specified neoplasms, NOS	477	18	15	18	20	0.5	0.5	0.6	0.7	2.32* (1.37, 3.29)
10. Unspecified malignant neoplasms	145	5	6	5	5	0.2	0.2	0.2	0.2	0.84 (−1.42, 3.16)

Abbreviations: ESR = European Standard Rates, CI = Confidence Interval, CNS = Central Nervous System, PNET = Primitive Neuro-Ectodermal Tumours, ICD-O = the International Classification of Diseases for Oncology. * Cancers in the Netherlands Cancer Registry are coded using the ICD-O valid at the time of diagnosis; 1st edition before 1993, 2nd edition between 1993 and 2000 and 3th edition since 2001. Cancer types were grouped based on the Surveillance, Epidemiology, and End Results Program

(SEER) AYA site recode adapted classification scheme. † Including specified low-grade astrocytic tumours, glioblastomas and anaplastic astrocytoma, and astrocytoma, NOS. § Including medulloblastomas and supratentorial PNET. ‡ Including unspecified malignant intracranial and intraspinal neoplasms and unspecified benign/border intracranial and intraspinal neoplasms. # Including carcinomas of the nasopharynx, other sites in the lip, oral cavity and pharynx, and nasal cavity, mid ear, sinuses, larynx and other ill-defined head and neck tumours. ¶ Including carcinomas of other ill-defined sites of the genitourinary tract, gastrointestinal tract, NOS, and adrenocortical carcinomas. ** Including Wilms tumours, neuroblastoma, other paediatric and embryonal tumours, NOS, paraganglioma and glomus tumours, other specified gonadal tumours, myeloma, mast cell, misc lymphoreticular neoplasms, NOS and other specified neoplasms, NOS. †† Incidence rates were calculated per 100,000 person-years using the mid-year population size as person-time denominator and standardized with weights from the 1976 European Standard Population. §§ AAPC and *p*-value outcomes denoted with "NA" could not be computed due to having zero counts in one or more individual years of diagnosis. * Indicates significant trends ($p < 0.05$).

Table S2. Total and average number of new cancer cases per year, incidence rates and average annual percentage change (AAPC) estimates of female adolescents and young adults (AYAs) aged 15−39 years at time of diagnosis in the Netherlands between 1990−2016.

Population Characteristics	Total Number of Cases	Average Number of New Cases per Year				Age-Standardised Incidence Rates (ESR) per 100,000 Person-Year††				AAPC (95% CI)§§
Period of Diagnosis		1990–2016	1990–1999	2000–2009	2010–2016	1990–2016	1990–1999	2000–2009	2010–2016	
All cancers	56,190	2,081	1,991	2,153	2,108	72.6	66.1	73.7	81.8	1.18* (1.03, 1.34)
Age (years)										
15–19	2,368	88	81	87	99	18.4	17.4	18.2	20.1	0.74* (0.20, 1.30)
20–24	4,013	149	142	146	162	28.6	25.6	30.2	31.2	1.03* (0.59, 1.48)
25–29	7,947	294	289	280	323	53.0	45.9	54.8	63.0	1.73* (1.45, 2.01)
30–34	15,614	578	570	589	576	99.7	90.3	101.1	114.2	1.34* (1.11, 1.57)
35–39	26,248	972	909	1,052	948	163.2	151.3	164.5	180.4	0.98* (0.79, 1.18)
All cancers, excluding gonadal germ cell tumours	55,668	2,062	1,973	2,134	2,086	71.9	65.5	73.0	80.9	1.18* (1.03, 1.33)
Age (years)										
15–19	2,272	84	78	83	94	17.5	16.7	17.5	19.2	0.74* (0.15, 1.33)
20–24	3,926	145	140	142	158	28.0	25.2	29.5	30.4	1.00* (0.56, 1.44)
25–29	7,832	290	285	276	318	52.2	45.3	54.0	62.1	1.74* (1.45, 2.02)
30–34	15,484	573	565	584	570	96.8	89.5	100.3	113.2	1.34* (1.11, 1.57)
35–39	26,154	969	906	1,049	944	163.5	150.8	163.9	179.7	0.98* (0.78, 1.18)
All cancers, excluding thyroid carcinomas	53,699	1,989	1,919	2,058	1,989	69.3	63.7	70.4	77.2	1.07* (0.92, 1.23)
Age (years)										
15–19	2,196	81	77	80	90	16.9	16.4	16.8	18.3	0.57* (0.05, 1.09)
20–24	3,718	138	133	136	148	26.6	23.9	28.2	28.4	0.53 (−0.28, 1.35)
25–29	7,447	276	272	262	301	49.7	43.3	51.2	58.7	1.66* (1.38, 1.95)
30–34	14,914	552	550	561	544	93.2	87.1	96.2	108.0	1.22* (0.99, 1.45)
35–39	25,424	942	888	1,020	907	158.9	147.8	159.4	172.5	0.88* (0.68, 1.08)

All cancers, excluding KS and NHL	54,395	2,015	1,925	2,084	2,043	70.2	63.9	71.3	79.2	1.19* (1.03, 1.35)
Age (years)										
15–19	2,198	81	76	80	90	16.9	16.3	16.9	18.4	0.61* (0.01, 1.21)
20–24	3,790	140	134	137	153	27.1	24.2	28.5	29.5	1.03* (0.59, 1.46)
25–29	7,630	283	277	267	312	50.9	44.1	52.3	60.9	1.75* (1.47, 2.04)
30–34	15,150	561	553	571	558	94.7	87.7	98.0	110.7	1.32* (1.09, 1.56)
35–39	25,627	949	884	1,028	929	160.2	147.2	160.7	176.8	1.02* (0.82, 1.21)
Cancer types*										
1. Leukaemia’s	1,528	57	58	59	51	2.3	2.0	2.2	1.9	0.58 (−0.25, 1.41)
1.1 Acute lymphoid leukaemia’s	404	15	15	16	14	0.6	0.5	0.6	0.5	0.52 (−0.86, 1.92)
1.2 Acute myeloid leukaemia’s	770	29	31	29	25	1.0	1.1	1.0	1.0	0.01 (−0.97, 1.00)
1.3 Chronic myeloid leukaemia’s	247	9	8	10	9	0.3	0.3	0.4	0.3	1.51 (−0.24, 3.30)
1.4 Other and unspecified leukaemia’s	107	4	3	5	4	0.1	0.1	0.2	0.2	2.38* (0.41, 4.40)
2. Lymphomas	4,251	157	150	158	167	6.7	5.2	5.9	6.2	1.35* (0.95, 1.74)
2.1 Non-Hodgkin lymphomas	1,746	65	63	66	64	2.3	2.1	2.4	2.5	2.28* (0.12, 4.49)
2.2 Hodgkin lymphomas	2,505	93	87	92	102	3.5	3.1	3.5	4.0	1.55* (0.97, 2.14)
3. CNS and other intracranial and intraspinal neoplasm	1,904	71	72	72	66	2.8	2.5	2.6	2.4	0.26 (−0.34, 0.86)
3.1 Astrocytoma’s†	1,146	42	45	43	39	1.5	1.5	1.5	1.5	−0.09 (−0.88, 0.71)
3.2 Other gliomas	390	14	15	15	13	0.5	0.5	0.5	0.5	0.44 (−0.78, 1.68)
3.3 Ependymomas	128	5	4	5	5	0.2	0.2	0.2	0.2	0.09 (−2.24, 2.47)
3.4 Medulloblastomas and other PNET§	84	3	3	4	2	0.1	0.1	0.1	0.1	0.87 (−2.24, 4.07)
3.5 Other specified intracranial and intraspinal neoplasms	40	1	2	1	2	0.1	0.1	0.0	0.1	NA
3.6 Unspecified intracranial and intraspinal neoplasms‡	116	4	4	4	6	0.2	0.1	0.1	0.2	3.00* (0.60, 5.45)
4. Osseous and Chondromatous neoplasms	602	22	21	23	24	1.0	0.7	0.9	0.9	0.89* (0.07, 1.72)
4.1 Osteosarcomas	215	8	8	8	8	0.3	0.3	0.3	0.3	−0.22 (−1.70, 1.29)
4.2 Chondrosarcomas	116	4	4	4	5	0.2	0.1	0.2	0.2	2.32* (0.36, 4.31)
4.3 Ewing tumours	178	7	6	7	7	0.3	0.2	0.3	0.3	0.95 (−0.63, 2.55)
4.4 Other specified and unspecified bone tumours	93	3	3	4	3	0.1	0.1	0.1	0.1	NA
5. Soft tissue sarcomas	1,589	59	64	60	51	2.3	2.2	2.2	1.8	−0.51 (−1.21, 0.19)
5.1 Fibromatous neoplasms	553	20	22	19	20	0.7	0.7	0.7	0.8	0.32 (−0.77, 1.42)

5.2 Rhabdomyosarcomas	77	3	4	2	3	0.1	0.1	0.1	0.1	NA
5.3.1.1 Specified soft tissue sarcomas (excluding Kaposi sarcoma)	782	29	31	31	22	1.0	1.1	1.1	0.9	−0.93 (−2.18, 0.33)
5.3.1.2 Kaposi sarcomas	49	2	2	3	1	0.1	0.1	0.1	0.0	NA
5.3.2 Unspecified soft tissue sarcomas	128	5	5	5	5	0.2	0.2	0.2	0.2	−0.35 (−2.04, 1.36)
6. Germ cell and Trophoblastic neoplasms	522	19	17	19	22	0.8	0.6	0.7	0.8	1.63* (0.70, 2.57)
6.1 Germ cell and trophoblastic neoplasms of the gonads	321	12	10	12	14	0.4	0.4	0.5	0.5	1.30 (0.02, 2.65)
6.2 Germ cell and trophoblastic neoplasms of non-gonadal sites	201	7	7	7	8	0.3	0.2	0.3	0.3	1.85* (0.30, 3.42)
7. Melanoma and skin carcinomas	10,882	403	341	450	424	14.4	11.5	15.9	15.0	1.91* (1.33, 2.50)
7.1 Melanoma	10,357	384	324	431	401	13.4	10.7	15.1	15.6	1.89* (1.27, 2.51)
7.2 Skin carcinomas	525	19	17	19	23	0.7	0.6	0.6	0.9	2.77* (1.29, 4.28)
8. Carcinomas	34,246	1,268	1,238	1,290	1,281	39.4	41.4	44.2	45.0	1.02* (0.67, 1.37)
8.1 Thyroid carcinomas	2,491	92	71	95	118	3.3	2.4	3.4	4.6	3.55* (3.04, 4.06)
8.2 Head and neck carcinomas#	754	28	27	30	26	1.0	0.9	1.0	1.0	−1.25 (−4.10, 1.68)
8.3 Carcinomas of trachea, bronchus, and lung	1,181	44	52	43	34	1.5	1.7	1.4	1.3	−1.17* (−1.80, −0.53)
8.4 Carcinomas of breast	18,128	671	626	706	686	22.8	20.5	23.2	26.5	1.35* (1.08, 1.63)
8.5.1 Carcinomas of kidney	377	14	12	13	18	0.5	0.4	0.5	0.7	3.01* (1.52, 4.51)
8.5.2 Carcinomas of bladder	184	7	6	8	6	0.2	0.2	0.3	0.3	0.77 (−1.51, 3.10)
8.5.3 Carcinomas of gonads	1,271	47	63	39	36	1.6	2.1	1.3	1.4	−2.35* (−3.85, −0.83)
8.5.4 Carcinomas of cervix and uterus	6,088	225	247	208	220	7.7	8.0	7.0	8.6	0.24 (−0.47, 0.96)
8.6.1 Carcinomas of colon/rectum	1,806	67	58	74	70	2.3	1.9	2.4	2.7	1.83* (1.18, 2.48)
8.6.2 Carcinomas of the stomach	423	16	16	16	14	0.5	0.5	0.6	0.5	0.38 (−1.01, 1.79)
8.6.3 Carcinomas of liver and intrahepatic bile ducts	109	4	4	4	5	0.1	0.1	0.2	0.2	2.54* (0.37, 4.76)
8.6.4 Carcinomas of pancreas	233	9	7	9	10	0.3	0.2	0.3	0.4	2.41* (0.61, 4.24)
8. Carcinomas of other ill-defined sites¶	1,201	44	48	46	37	1.5	1.6	1.5	1.5	−0.48 (−1.20, 0.24)

9. Miscellaneous specified neoplasms, NOS	580	21	24	19	20	0.7	0.8	0.7	0.7	−0.13 (−1.41, 1.16)
10. Unspecified malignant neoplasms	86	3	5	3	1	0.1	0.2	0.1	0.0	NA

Abbreviations:ESR = European Standard Rates, CI = Confidence Interval, CNS = Central Nervous System, PNET = Primitive Neuro-Ectodermal Tumours, ICD-O = the International Classification of Diseases for Oncology. * Cancers in the Netherlands Cancer Registry are coded using the ICD-O valid at the time of diagnosis; 1st edition before 1993, 2nd edition between 1993 and 2000 and 3th edition since 2001. Cancer types were grouped based on the Surveillance, Epidemiology, and End Results Program (SEER) AYA site recode adapted classification scheme. † Including specified low-grade astrocytic tumours, glioblastomas and anaplastic astrocytoma, and astrocytoma, NOS. § Including medulloblastomas and supratentorial PNET. ‡ Including unspecified malignant intracranial and intraspinal neoplasms and unspecified benign/border intracranial and intraspinal neoplasms. # Including carcinomas of the nasopharynx, other sites in the lip, oral cavity and pharynx, and nasal cavity, mid ear, sinuses, larynx and other ill-defined head and neck tumours. ¶ Including carcinomas of other ill-defined sites of the genitourinary tract, gastrointestinal tract, NOS, and adrenocortical carcinomas. ** Including Wilms tumours, neuroblastoma, other paediatric and embryonal tumours, NOS, paraganglioma and glomus tumours, other specified gonadal tumours, myeloma, mast cell, misc lymphoreticular neoplasms, NOS and other specified neoplasms, NOS. †† Incidence rates were calculated per 100,000 person-years using the mid-year population size as person-time denominator and standardized with weights from the 1976 European Standard Population. §§ AAPC and p-value outcomes denoted with ″NA″ could not be computed due to having zero counts in one or more individual years of diagnosis. * Indicates significant trends ($p < 0.05$).

Table S3. Number at risk and five-year relative survival estimates with 95% confidence intervals of male adolescent and young adult (AYA) cancer patients aged 15−39 years at time of diagnosis in the Netherlands between 1990−2016 and presented by age, cancer type and period of diagnosis.

**Population Characteristics**	**Period of Diagnosis**								***p*-Value ††**
	**1990–2016**		**1990–1999**		**2000–2009**		**2010–2016**		
	N at risk	% RS (95% CI)	N at risk	% RS (95% CI)	N at risk	% RS (95% CI)	N at risk	% RS (95% CI)	
All cancers	39,038	78.1 (77.7, 78.5)	13,481	70.0 (69.2, 70.8)	14,873	80.2 (79.5, 80.8)	10,684	85.7 (85.0, 86.4)	0.00
Age (years)									
15–19	3,066	79.0 (77.5, 80.4)	1,011	70.0 (67.0, 72.7)	1,173	80.9 (78.5, 83.0)	882	87.2 (84.8, 89.4)	0.00
20–24	4,961	83.4 (82.3, 84.4)	1,700	76.4 (74.3, 78.3)	1,809	85.3 (83.6, 86.9)	1,452	89.4 (87.6, 91.0)	0.00
25–29	7,474	83.1 (82.2, 84.0)	2,653	76.7 (75.1, 78.3)	2,679	84.9 (83.4, 86.2)	2,142	89.4 (87.9, 90.7)	0.00
30–34	9,987	79.8 (79.0, 80.6)	3,514	71.7 (70.1, 73.2)	3,742	82.3 (81.0, 83.5)	2,731	87.2 (85.8, 88.5)	0.00
35–39	13,550	71.9 (71.1, 72.6)	4,603	62.5 (61.0, 63.9)	5,470	74.5 (73.3, 75.7)	3,477	80.5 (79.1, 81.9)	0.00
All cancers, excluding gonadal germ cell tumours	27,940	70.6 (70.0, 71.1)	10,324	62.2 (61.2, 63.1)	10,516	73.2 (72.3, 74.0)	7,100	79.3 (78.3, 80.2)	0.00
Age (years)									
15–19	2,398	75.0 (73.2, 76.7)	802	65.1 (61.7, 68.4)	900	76.8 (73.9, 79.4)	696	84.4 (81.3, 87.0)	0.00
20–24	3,051	75.0 (73.4, 76.5)	1,150	67.4 (64.6, 70.1)	1,068	77.3 (74.6, 79.7)	833	82.9 (80.0, 85.4)	0.00
25–29	4,560	74.3 (73.0, 75.6)	1,820	68.2 (66.0, 70.3)	1,540	76.0 (73.8, 78.1)	1,200	82.2 (79.8, 84.4)	0.00
30–34	6,860	71.6 (70.5, 72.7)	2,598	62.6 (60.7, 64.5)	2,538	74.9 (73.1, 76.5)	1,724	80.5 (78.4, 82.4)	0.00
35–39	11,071	66.2 (65.3, 67.1)	3,954	57.0 (55.4, 58.5)	4,470	69.5 (68.1, 70.8)	2,647	74.7 (72.9, 76.4)	0.00

All cancers, excluding thyroid carcinomas	38,231	77.7 (77.3, 78.1)	13,221	69.5 (68.7, 70.3)	14,579	79.8 (79.2, 80.5)	10,431	85.5 (84.7, 86.2)	0.00
Age (years)									
15–19	3,004	78.6 (77.1, 80.0)	992	69.4 (66.4, 72.2)	1,151	80.6 (78.2, 82.8)	861	86.9 (84.4, 89.1)	0.00
20–24	4,873	83.2 (82.1, 84.2)	1,666	76.1 (73.9, 78.1)	1,783	85.2 (83.4, 86.8)	1,424	89.3 (87.4, 90.9)	0.00
25–29	7,299	82.8 (81.9, 83.7)	2,592	76.3 (74.6, 77.9)	2,621	84.6 (83.2, 86.0)	2,086	89.1 (87.6, 90.5)	0.00
30–34	9,778	79.5 (78.7, 80.3)	3,451	71.2 (69.7, 72.7)	3,670	82.1 (80.8, 83.3)	265	74.0 (72.8, 75.2)	0.00
35–39	13,277	71.4 (70.6, 72.1)	4,520	61.9 (60.4, 63.3)	5,354	87.0 (85.6, 88.3)	3,403	80.1 (78.7, 81.5)	0.00
All cancers, excluding KS and NHL	35,403	78.9 (78.4, 79.3)	11,935	72.2 (71.4, 73.0)	13,597	80.1 (79.4, 80.8)	9,871	85.6 (84.8, 86.3)	0.00
Age (years)									
15–19	2,789	79.1 (77.5, 80.6)	916	70.8 (67.7, 73.7)	1,075	80.5 (78.0, 82.8)	798	87.0 (84.4, 89.3)	0.00
20–24	4,590	84.1 (83.0, 85.1)	1,565	77.8 (75.7, 79.8)	1,681	85.5 (83.7, 87.2)	1,344	89.8 (87.9, 91.4)	0.00
25–29	6,862	84.2 (83.2, 85.0)	2,379	79.1 (77.4, 80.7)	2,476	85.3 (83.8, 86.7)	2,007	89.2 (87.7, 90.6)	0.00
30–34	8,984	81.4 (80.6, 82.2)	3,037	75.5 (73.9, 77.0)	3,426	82.6 (81.3, 83.9)	2,521	87.2 (85.7, 88.5)	0.00
35–39	12,178	72.0 (71.2, 72.8)	4,038	63.8 (62.3, 65.3)	4,939	73.7 (72.5, 75.0)	3,201	79.9 (78.4, 81.4)	0.00
Cancer types*									
1. Leukaemia’s	2,028	59.6 (57.4, 61.8)	757	46.4 (42.7, 49.9)	730	62.9 (59.3, 66.3)	541	74.3 (70.2, 77.9)	0.00
1.1 Acute lymphoid leukaemia’s	664	55.6 (51.7, 59.4)	244	42.8 (36.5, 49.0)	235	57.9 (51.3, 64.0)	185	70.1 (62.6, 76.4)	0.00
1.2 Acute myeloid leukaemia’s	732	47.2 (43.5, 50.8)	305	37.9 (32.4, 43.3)	248	48.2 (41.8, 54.3)	179	61.9 (54.1, 68.8)	0.00
1.3 Chronic myeloid leukaemia’s	385	77.5 (72.9, 81.4)	128	50.9 (41.8, 59.2)	150	88.3 (81.9, 92.6)	107	94.9 (87.6, 98.1)	0.00
1.4 Other and unspecified leukaemia’s	247	79.5 (73.7, 84.1)	80	82.7 (72.2, 89.6)	97	73.2 (63.1, 81.0)	70	85.1 (73.7, 91.9)	0.76
2. Lymphomas	5,960	83.9 (82.9, 84.9)	2,176	75.7 (73.8, 77.4)	2,258	86.6 (85.1, 87.9)	1,526	92.1 (90.6, 93.4)	0.00
2.1 Non-Hodgkin lymphomas	2,994	75.1 (73.5, 76.6)	1,113	62.1 (59.2, 64.9)	1,134	80.1 (77.6, 82.3)	747	87.1 (84.4, 89.4)	0.00
2.2 Hodgkin lymphomas	2,966	92.8 (91.8, 93.7)	1,063	89.8 (87.8, 91.6)	1,124	93.1 (91.4, 94.4)	779	96.9 (95.3, 98.1)	0.00
3. CNS and other intracranial and intraspinal neoplasm	2,740	57.4 (55.5, 59.2)	1,057	53.4 (50.4, 56.4)	991	56.3 (53.1, 59.3)	692	65.2 (61.2, 68.8)	0.00
3.1 Astrocytoma’s†	1,696	49.2 (46.7, 51.6)	662	46.2 (42.4, 50.0)	601	47.1 (43.0, 51.0)	433	56.7 (51.4, 61.6)	0.00
3.2 Other gliomas	548	69.2 (65.0, 72.9)	206	63.9 (56.9, 70.1)	216	68.7 (62.0, 74.5)	126	79.2 (70.6, 85.6)	0.00
3.3 Ependymomas	171	84.9 (78.4, 89.6)	72	82.3 (71.3, 89.5)	59	90.2 (79.1, 95.7)	40	79.6 (61.2, 90.0)	0.74
3.4 Medulloblastomas and other PNET§	135	69.1 (60.3, 76.3)	52	71.1 (56.5, 81.6)	49	63.4 (48.3, 75.2)	34	74.5 (54.5, 86.8)	0.95
3.5 Other specified intracranial and intraspinal neoplasms	41	51.4 (35.2, 65.4)	17	59.1 (32.7, 78.2)	12	33.4 (10.3, 59.0)	12	58.5 (27.1, 80.3)	0.83
3.6 Unspecified intracranial and intraspinal neoplasms‡	149	66.3 (57.9, 73.5)	48	43.0 (28.8, 56.6)	54	70.6 (56.4, 80.9)	47	85.3 (69.2, 93.4)	0.00
4. Osseous and Chondromatous neoplasms	832	61.6 (58.1, 64.8)	308	54.4 (48.6, 59.8)	305	60.7 (55.0, 66.0)	219	73.5 (66.6, 79.3)	0.00
4.1 Osteosarcomas	332	55.4 (49.7, 60.7)	133	48.3 (39.6, 56.5)	113	53.2 (43.6, 62.0)	86	71.6 (59.8, 80.5)	0.00
4.2 Chondrosarcomas	140	85.7 (78.6, 90.7)	50	78.3 (64.0, 87.5)	49	84.0 (70.2, 91.8)	41	97.8 (84.1, 99.9)	0.01
4.3 Ewing tumours	275	49.5 (43.4, 55.4)	97	42.3 (32.4, 52.0)	107	50.2 (40.3, 59.3)	71	56.8 (43.0, 68.4)	0.03
4.4 Other specified and unspecified bone tumours	85	84.6 (74.8, 90.9)	28	82.5 (62.6, 92.6)	36	83.6 (66.9, 92.5)	21	88.9 (61.5, 97.4)	0.54
5. Soft tissue sarcomas	2,214	64.1 (62.0, 66.1)	1,073	53.2 (50.1, 56.2)	745	74.5 (71.2, 77.5)	396	74.1 (69.1, 78.4)	0.00

5.1 Fibromatous neoplasms	504	93.2 (90.5, 95.2)	240	89.9 (85.3, 93.2)	186	95.4 (91.0, 97.7)	78	99.0 (91.4, 100.1)	0.00
5.2 Rhabdomyosarcomas	125	36.4 (27.9, 44.9)	49	28.7 (16.9, 41.6)	44	41.0 (26.5, 55.0)	32	43.2 (25.7, 59.5)	0.23
5.3.1.1 Specified soft tissue sarcomas (excluding Kaposi sarcoma)	817	65.1 (61.6, 68.3)	308	63.4 (57.7, 68.5)	316	66.6 (61.0, 71.5)	193	64.3 (56.4, 71.1)	0.63
5.3.1.2 Kaposi sarcomas	641	48.6 (44.6, 52.5)	433	29.2 (25.0, 33.6)	142	87.7 (80.9, 92.2)	66	95.7 (86.7, 98.8)	0.00
5.3.2 Unspecified soft tissue sarcomas	127	47.3 (38.3, 55.8)	43	43.3 (28.2, 57.6)	57	45.0 (31.7, 57.4)	27	58.8 (37.9, 74.8)	0.21
6. Germ cell and Trophoblastic neoplasms	11,098	97.1 (96.7, 97.4)	3,157	95.5 (94.7, 96.2)	4,357	97.0 (96.5, 97.5)	3,584	98.6 (98.1, 99.0)	0.00
6.1 Germ cell and trophoblastic neoplasms of the gonads	10,755	97.7 (97.4, 98.0)	3,020	96.5 (95.8, 97.2)	4,222	97.8 (97.2, 98.2)	3,513	98.9 (98.4, 99.2)	0.00
6.2 Germ cell and trophoblastic neoplasms of non-gonadal sites	343	76.1 (71.2, 80.4)	137	71.8 (63.4, 78.6)	135	74.2 (65.9, 80.8)	71	87.9 (76.9, 94.0)	0.01
7. Melanoma and skin carcinomas	6,087	88.0 (87.2, 88.9)	1,986	84.3 (82.6, 85.8)	2,417	88.6 (87.2, 89.8)	1,684	92.3 (90.8, 93.6)	0.00
7.1 Melanoma	5,630	87.5 (86.6, 88.4)	1,842	83.7 (81.9, 85.4)	2,242	88.0 (86.6, 89.3)	1,546	92.0 (90.4, 93.4)	0.00
7.2 Skin carcinomas	457	94.0 (91.3, 96.0)	144	91.3 (85.1, 95.1)	175	95.8 (91.4, 98.1)	138	95.0 (89.4, 97.8)	0.24
8. Carcinomas	7,457	57.4 (56.3, 58.5)	2,757	49.2 (47.3, 51.0)	2,836	59.7 (57.9, 61.5)	1,864	66.1 (63.8, 68.3)	0.00
8.1 Thyroid carcinomas	807	96.0 (94.3, 97.2)	260	95.1 (91.5, 97.2)	294	95.9 (92.8, 97.8)	253	97.1 (93.8, 98.8)	0.25
8.2 Head and neck carcinomas#	1,077	77.3 (74.6, 79.7)	408	69.3 (64.6, 73.6)	429	81.3 (77.2, 84.7)	240	84.1 (78.5, 88.3)	0.00
8.3 Carcinomas of trachea, bronchus, and lung	963	26.7 (23.9, 29.6)	389	19.9 (16.1, 24.1)	360	29.2 (24.5, 34.0)	214	34.7 (28.1, 41.4)	0.00
8.4 Carcinomas of breast	37	77.9 (60.4, 88.5)	11	82.2 (45.0, 95.6)	19	68.7 (43.0, 84.8)	7	100.4 (100.4, 100.4)	0.70
8.5.1 Carcinomas of kidney	605	78.2 (74.6, 81.4)	184	67.1 (59.7, 73.4)	249	80.5 (74.9, 85.0)	172	88.0 (82.1, 92.1)	0.00
8.5.2 Carcinomas of bladder	255	64.9 (58.6, 70.5)	106	69.8 (59.9, 77.7)	92	64.4 (53.7, 73.3)	57	55.4 (40.7, 67.9)	0.08
8.5.3 Carcinomas of gonads	8	87.8 (38.8, 98.5)	2	50.2 (0.6, 91.5)	5	100.3 (100.3, 100.3)	1	100.4 (100.4, 100.4)	1.00
8.5.4 Carcinomas of cervix and uterus									
8.6.1 Carcinomas of colon/rectum	1,823	64.8 (62.5, 67.0)	602	59.6 (55.6, 63.5)	713	66.6 (63.0, 70.0)	508	68.3 (63.9, 72.4)	0.00
8.6.2 Carcinomas of the stomach	516	28.5 (24.6, 32.6)	223	23.3 (18.0, 29.1)	179	30.7 (24.1, 37.7)	114	35.7 (26.6, 44.8)	0.02
8.6.3 Carcinomas of liver and intrahepatic bile ducts	150	23.3 (16.6, 30.7)	54	10.6 (4.0, 21.0)	52	20.7 (10.8, 32.9)	44	41.6 (26.2, 56.4)	0.00
8.6.4 Carcinomas of pancreas	199	23.1 (17.5, 29.2)	71	14.2 (7.3, 23.3)	78	18.0 (10.4, 27.3)	50	43.5 (29.4, 56.8)	0.00
8. Carcinomas of other ill-defined sites¶	1,017	32.6 (29.7, 35.5)	447	25.1 (21.1, 29.2)	366	34.1 (29.2, 39.0)	204	46.5 (39.4, 53.3)	0.00
9. Miscellaneous specified neoplasms, NOS	477	66.5 (62.0, 70.7)	154	52.1 (43.8, 59.7)	181	68.7 (61.4, 75.0)	142	80.8 (72.9, 86.7)	0.00
10. Unspecified malignant neoplasms	145	33.5 (25.9, 41.3)	56	16.1 (8.0, 26.9)	53	39.5 (26.3, 52.3)	36	51.6 (33.9, 66.8)	0.00

Abbreviations: RS = Relative Survival, CI = Confidence Intervals, CNS= Central Nervous System, PNET = Primitive Neuro-Ectodermal Tumours, ICD-O = the International Classification of Diseases for Oncology. * Cancers in the Netherlands Cancer Registry are coded using the ICD-O valid at the time of diagnosis; 1st edition before 1993, 2nd edition between 1993 and 2000 and 3th edition since 2001. Cancer types were grouped based on the Surveillance, Epidemiology, and End Results Program (SEER) AYA site recode adapted classification scheme. † Including specified low-grade astrocytic tumours, glioblastomas and anaplastic astrocytoma, and astrocytoma, NOS. § Including medulloblastomas and supratentorial PNET. ‡ Including unspecified malignant intracranial and intraspinal neoplasms and unspecified benign/border intracranial and intraspinal neoplasms. # Including carcinomas of the nasopharynx, other sites in the lip, oral cavity and pharynx, and nasal cavity, mid ear, sinuses, larynx and other ill-defined head and neck tumours. ¶ Including carcinomas of other ill-defined sites of the genitourinary tract, gastrointestinal tract, NOS, and adrenocortical carcinomas. ** Including Wilms tumours, neuroblastoma, other paediatric and embryonal tumours, NOS, paraganglioma and glomus tumours,

other specified gonadal tumours, myeloma, mast cell, misc lymphoreticular neoplasms, NOS and other specified neoplasms, NOS. ✝✝ P-values were obtained from a likelihood ratio test comparing a generalized linear model including the midpoint of the calendar period and a model without calendar period. All models assumed a Poisson distribution for the observed number of deaths. Significance was determined if $p < 0.05$.

Table S4. Number at risk and five-year relative survival estimates with 95% confidence intervals of female adolescent and young adult (AYA) cancer patients aged 15−39 years at time of diagnosis in the Netherlands between 1990–2016 and presented by age, cancer type and period of diagnosis.

Population Characteristics	Period of Diagnosis								*p*-Value ✝✝
	1990–2016		1990–1999		2000–2009		2010–2016		
	N at risk	% RS (95% CI)	N at risk	% RS (95% CI)	N at risk	% RS (95% CI)	N at risk	% RS (95% CI)	
All cancers	56,190	81.2 (80.8, 81.5)	19,905	76.2 (75.6, 76.8)	21,532	82.2 (81.6, 82.7)	14,753	86.9 (86.3, 87.5)	0.00
Age (years)									
15–19	2,368	82.4 (80.8, 83.9)	813	78.3 (75.2, 81.0)	865	83.3 (80.6, 85.6)	690	85.9 (83.0, 88.4)	0.00
20–24	4,013	85.0 (83.9, 86.1)	1,423	79.8 (77.6, 81.8)	1,455	85.9 (84.0, 87.6)	1,135	90.6 (88.6, 92.3)	0.00
25–29	7,947	83.3 (82.4, 84.1)	2,887	78.9 (77.3, 80.3)	2,798	83.5 (82.0, 84.8)	2,262	89.1 (87.7, 90.4)	0.00
30–34	15,614	81.7 (81.1, 82.3)	5,695	76.7 (75.6, 77.8)	5,890	83.0 (82.0, 83.9)	4,029	87.3 (86.1, 88.3)	0.00
35–39	26,248	79.5 (79.0, 80.0)	9,087	74.2 (73.3, 75.1)	10,524	80.7 (80.0, 81.5)	6,637	85.4 (84.5, 86.3)	0.00
All cancers, excluding gonadal germ cell tumours	55,668	81.1 (80.7, 81.4)	19,731	76.1 (75.4, 76.7)	21,338	82.1 (81.5, 82.6)	14,599	86.8 (86.2, 87.4)	0.00
Age (years)									
15–19	2,272	81.8 (80.1, 83.3)	780	77.6 (74.5, 80.4)	833	82.8 (80.0, 85.2)	659	85.3 (82.2, 87.9)	0.00
20–24	3,926	84.8 (83.6, 85.9)	1,401	79.5 (77.3, 81.5)	1,418	85.7 (83.8, 87.4)	1,107	90.4 (88.3, 92.1)	0.00
25–29	7,832	83.2 (82.3, 84.0)	2,845	78.8 (77.3, 80.3)	2,758	83.4 (82.0, 84.8)	2,229	89.0 (87.5, 90.3)	0.00
30–34	15,484	81.6 (80.9, 82.2)	5,648	76.6 (75.5, 77.7)	5,843	82.9 (81.9, 83.8)	3,993	87.2 (86.0, 88.3)	0.00
35–39	26,154	79.5 (79.0, 80.0)	9,057	74.2 (73.2, 75.1)	10,486	80.7 (79.9, 81.5)	6,611	85.4 (84.4, 86.3)	0.00
All cancers, excluding thyroid carcinomas	53,699	80.3 (80.0, 80.7)	19,191	75.3 (74.7, 75.9)	20,582	81.4 (80.8, 81.9)	13,926	86.2 (85.6, 86.8)	0.00
Age (years)									
15–19	2,196	81.0 (79.3, 82.6)	768	77.0 (73.8, 79.8)	799	81.9 (79.1, 84.4)	629	84.5 (81.3, 87.3)	0.00
20–24	3,718	83.9 (82.6, 85.0)	1,326	78.4 (76.0, 80.5)	1,358	84.9 (82.9, 86.7)	1,034	89.7 (87.5, 91.5)	0.00
25–29	7,447	82.2 (81.3, 83.1)	2,723	77.7 (76.1, 79.2)	2,616	82.4 (80.8, 83.8)	2,108	88.4 (86.9, 89.8)	0.00
30–34	14,914	80.9 (80.2, 81.5)	5,497	75.9 (74.7, 77.0)	5,608	82.2 (81.1, 83.2)	3,809	86.7 (85.5, 87.8)	0.00
35–39	25,424	78.9 (78.4, 79.4)	8,877	73.6 (72.7, 74.5)	10,201	80.2 (79.4, 80.9)	6,346	84.8 (83.8, 85.7)	0.00
All cancers, excluding KS and NHL	54,395	81.3 (80.9, 81.6)	19,254	76.4 (75.8, 77.0)	20,843	82.3 (81.7, 82.8)	14,298	86.8 (86.2, 87.4)	0.00
Age (years)									
15–19	2,198	82.8 (81.1, 84.3)	763	79.1 (76.0, 81.8)	804	83.6 (80.9, 86.0)	631	86.0 (82.9, 88.6)	0.00
20–24	3,790	85.5 (84.3, 86.6)	1,343	80.6 (78.3, 82.6)	1,373	86.4 (84.5, 88.1)	1,074	90.7 (88.6, 92.4)	0.00
25–29	7,630	83.5 (82.6, 84.3)	2,773	79.3 (77.7, 80.7)	2,671	83.6 (82.1, 85.0)	2,186	89.3 (87.8, 90.6)	0.00
30–34	15,150	81.9 (81.2, 82.5)	5,534	77.1 (75.9, 78.2)	5,712	83.2 (82.2, 84.1)	3,904	87.2 (86.1, 88.3)	0.00
35–39	25,627	79.5 (79.0, 80.0)	8,841	74.2 (73.2, 75.1)	10,283	80.8 (80.0, 81.5)	6,503	85.2 (84.3, 86.1)	0.00
Cancer types *									

1. Leukaemia's	1,528	58.6 (56.0, 61.0)	577	48.0 (43.8, 52.0)	591	61.5 (57.5, 65.3)	360	70.9 (65.7, 75.5)	0.00
1.1 Acute lymphoid leukaemia's	404	57.2 (52.1, 61.9)	152	44.6 (36.6, 52.3)	156	59.1 (50.9, 66.3)	96	75.2 (64.8, 82.9)	0.00
1.2 Acute myeloid leukaemia's	770	50.6 (46.9, 54.1)	311	41.8 (36.3, 47.3)	285	52.7 (46.7, 58.3)	174	62.7 (54.8, 69.6)	0.00
1.3 Chronic myeloid leukaemia's	247	80.1 (74.5, 84.6)	83	67.7 (56.4, 76.6)	104	84.8 (76.3, 90.5)	60	89.3 (77.5, 95.2)	0.00
1.4 Other and unspecified leukaemia's	107	71.5 (61.7, 79.2)	31	73.8 (54.1, 86.1)	46	72.0 (56.5, 82.8)	30	68.1 (47.2, 82.2)	0.63
2. Lymphomas	4,251	88.2 (87.2, 89.2)	1,504	83.2 (81.2, 85.0)	1,579	88.9 (87.2, 90.4)	1,168	94.2 (92.6, 95.5)	0.00
2.1 Non-Hodgkin lymphomas	1,746	77.9 (75.8, 79.8)	632	70.2 (66.4, 73.6)	663	78.4 (75.0, 81.3)	451	88.4 (84.9, 91.1)	0.00
2.2 Hodgkin lymphomas	2,505	95.5 (94.5, 96.2)	872	92.6 (90.6, 94.2)	916	96.5 (95.0, 97.5)	717	97.8 (96.2, 98.8)	0.00
3. CNS and other intracranial and intraspinal neoplasm	1,904	60.8 (58.5, 63.0)	723	55.5 (51.8, 59.1)	716	59.6 (55.9, 63.1)	465	71.7 (67.0, 75.9)	0.00
3.1 Astrocytoma's †	1,146	55.1 (52.1, 58.0)	448	50.7 (46.0, 55.3)	426	53.1 (48.2, 57.7)	272	66.1 (59.6, 71.8)	0.00
3.2 Other gliomas	390	66.7 (61.7, 71.2)	149	62.6 (54.3, 69.9)	151	65.0 (56.8, 72.0)	90	78.2 (67.3, 85.8)	0.03
3.3 Ependymomas	128	83.1 (75.1, 88.7)	42	78.3 (62.3, 88.2)	51	82.6 (69.0, 90.6)	35	90.8 (73.8, 97.1)	0.18
3.4 Medulloblastomas and other PNE §	84	56.1 (44.7, 66.1)	30	55.4 (35.8, 71.2)	37	54.2 (37.0, 68.5)	17	60.4 (31.3, 80.4)	0.78
3.5 Other specified intracranial and intraspinal neoplasms	40	72.1 (55.1, 83.6)	17	65.0 (37.9, 82.7)	12	83.5 (48.3, 95.8)	11	65.6 (25.1, 88.0)	0.48
3.6 Unspecified intracranial and intraspinal neoplasms ‡	116	73.4 (64.1, 80.7)	37	56.0 (38.5, 70.4)	39	77.1 (60.5, 87.5)	40	86.7 (70.4, 94.4)	0.00
4. Osseous and Chondromatous neoplasms	602	67.6 (63.6, 71.2)	207	62.2 (55.2, 68.5)	230	68.2 (61.7, 73.8)	165	72.7 (64.5, 79.2)	0.01
4.1 Osteosarcomas	215	70.1 (63.4, 75.8)	78	70.4 (58.8, 79.3)	81	71.4 (60.1, 80.0)	56	66.6 (51.4, 78.0)	0.84
4.2 Chondrosarcomas	116	77.9 (69.0, 84.6)	38	71.2 (54.0, 83.0)	41	80.4 (64.4, 89.8)	37	82.5 (64.6, 91.9)	0.23
4.3 Ewing tumours	178	47.6 (40.0, 54.8)	59	33.3 (21.6, 45.4)	71	46.6 (34.7, 57.6)	48	66.3 (50.1, 78.3)	0.00
4.4 Other specified and unspecified bone tumours	93	87.0 (78.1, 92.5)	32	84.6 (66.6, 93.4)	37	89.4 (73.9, 96.0)	24	87.6 (66.2, 95.9)	0.83
5. Soft tissue sarcomas	1,589	76.6 (74.4, 78.7)	636	72.2 (68.5, 75.6)	599	78.4 (74.9, 81.5)	354	81.7 (77.0, 85.5)	0.00
5.1 Fibromatous neoplasms	553	96.4 (94.3, 97.7)	220	93.4 (89.1, 96.1)	192	98.1 (94.7, 99.5)	141	98.8 (94.6, 99.8)	0.00
5.2 Rhabdomyosarcomas	77	47.8 (36.2, 58.5)	35	45.8 (29.0, 61.2)	22	45.5 (24.5, 64.4)	20	50.6 (25.5, 71.3)	0.45
5.3.1.1 Specified soft tissue sarcomas (excluding Kaposi sarcoma)	782	70.6 (67.2, 73.7)	311	67.3 (61.7, 72.2)	314	71.5 (66.1, 76.2)	157	76.1 (68.4, 82.3)	0.05
5.3.1.2 Kaposi sarcomas	49	72.9 (57.8, 83.3)	19	45.2 (22.1, 65.8)	26	88.4 (68.0, 96.2)	4	100.1 (100.1, 100.1)	0.00
5.3.2 Unspecified soft tissue sarcomas	128	47.3 (38.3, 55.7)	51	39.3 (26.0, 52.4)	45	53.1 (37.5, 66.5)	32	52.0 (33.1, 68.0)	0.32
6. Germ cell and Trophoblastic neoplasms	522	92.1 (89.4, 94.1)	174	88.2 (82.3, 92.2)	194	92.4 (87.6, 95.4)	154	96.1 (91.4, 98.3)	0.01
6.1 Germ cell and trophoblastic neoplasms of the gonads	321	92.6 (89.1, 95.1)	103	88.6 (80.6, 93.4)	123	92.8 (86.5, 96.3)	95	96.9 (90.6, 99.1)	0.03
6.2 Germ cell and trophoblastic neoplasms of non-gonadal sites	201	91.2 (86.3, 94.5)	71	87.6 (77.3, 93.5)	71	91.8 (82.3, 96.3)	59	94.8 (84.3, 98.4)	0.12
7. Melanoma and skin carcinomas	10,882	95.0 (94.5, 95.4)	3,414	93.2 (92.3, 94.1)	4,499	95.2 (94.6, 95.8)	2,969	96.7 (95.9, 97.4)	0.00
7.1 Melanoma	10,357	94.9 (94.4, 95.3)	3,242	93.1 (92.2, 94.0)	4,308	95.1 (94.4, 95.8)	2,807	96.7 (95.9, 97.4)	0.00
7.2 Skin carcinomas	525	96.6 (94.5, 97.9)	172	95.7 (91.2, 98.0)	191	97.1 (93.4, 98.9)	162	96.8 (92.0, 98.8)	0.43
8. Carcinomas	34,246	78.5 (78.1, 79.0)	12,382	73.6 (72.8, 74.4)	12,899	79.5 (78.8, 80.2)	8,965	84.6 (83.8, 85.4)	0.00

8.1 Thyroid carcinomas	2,491	99.0 (98.5, 99.4)	714	99.2 (98.0, 99.7)	950	99.2 (98.3, 99.7)	827	98.7 (97.5, 99.3)	0.39
8.2 Head and neck carcinomas #	754	82.4 (79.4, 84.9)	272	80.1 (74.8, 84.4)	298	82.0 (77.1, 86.0)	184	86.4 (80.3, 90.8)	0.08
8.3 Carcinomas of trachea, bronchus, and lung	1,181	31.1 (28.4, 33.7)	516	27.4 (23.6, 31.3)	425	29.8 (25.5, 34.2)	240	40.8 (34.1, 47.3)	0.00
8.4 Carcinomas of breast	18,128	82.6 (82.1, 83.2)	6,264	76.0 (74.9, 77.0)	7,064	84.7 (83.9, 85.6)	4,800	89.4 (88.3, 90.3)	0.00
8.5.1 Carcinomas of kidney	377	82.3 (78.0, 85.8)	121	78.8 (70.3, 85.1)	133	83.0 (75.4, 88.4)	123	84.7 (76.6, 90.2)	0.18
8.5.2 Carcinomas of bladder	184	48.5 (41.0, 55.6)	63	55.4 (42.2, 66.8)	76	40.9 (29.8, 51.7)	45	52.4 (36.7, 66.0)	0.40
8.5.3 Carcinomas of gonads	1,271	66.5 (63.7, 69.0)	633	70.0 (66.2, 73.4)	387	62.4 (57.3, 67.1)	251	62.3 (55.3, 68.6)	0.06
8.5.4 Carcinomas of cervix and uterus	6,088	87.2 (86.3, 88.0)	2,466	86.3 (84.9, 87.7)	2,079	86.7 (85.1, 88.1)	1,543	89.3 (87.5, 90.9)	0.01
8.6.1 Carcinomas of colon/rectum	1,806	63.4 (61.1, 65.7)	580	58.7 (54.6, 62.6)	737	65.0 (61.4, 68.3)	489	67.1 (62.4, 71.3)	0.00
8.6.2 Carcinomas of the stomach	423	26.1 (21.9, 30.5)	162	19.6 (13.9, 26.1)	164	30.3 (23.4, 37.6)	97	31.4 (22.3, 40.9)	0.05
8.6.3 Carcinomas of liver and intrahepatic bile ducts	109	31.1 (22.3, 40.2)	35	27.0 (13.5, 42.5)	42	28.6 (16.0, 42.6)	32	37.3 (18.7, 56.1)	0.13
8.6.4 Carcinomas of pancreas	233	34.9 (28.8, 41.1)	73	31.6 (21.3, 42.4)	88	24.4 (16.0, 33.9)	72	50.8 (38.6, 61.8)	0.01
8. Carcinomas of other ill-defined sites¶	1,201	44.9 (42.0, 47.7)	483	37.3 (33.0, 41.6)	456	45.1 (40.5, 49.6)	262	58.3 (51.7, 64.4)	0.00
9. Miscellaneous specified neoplasms, NOS	580	75.5 (71.8, 78.9)	243	75.1 (69.1, 80.1)	194	74.9 (68.2, 80.5)	143	77.3 (69.1, 83.6)	0.73
10. Unspecified malignant neoplasms	86	30.0 (20.6, 39.9)	45	26.8 (14.9, 40.2)	31	28.5 (14.0, 44.9)	10	50.2 (18.4, 75.6)	0.31

Abbreviations: RS = Relative Survival, CI = Confidence Intervals, CNS= Central Nervous System, PNET = Primitive Neuro-Ectodermal Tumours, ICD-O = the International Classification of Diseases for Oncology. * Cancers in the Netherlands Cancer Registry are coded using the ICD-O valid at the time of diagnosis; 1st edition before 1993, 2nd edition between 1993 and 2000 and 3th edition since 2001. Cancer types were grouped based on the Surveillance, Epidemiology, and End Results Program (SEER) AYA site recode adapted classification scheme. † Including specified low-grade astrocytic tumours, glioblastomas and anaplastic astrocytoma, and astrocytoma, NOS. § Including medulloblastomas and supratentorial PNET. ‡ Including unspecified malignant intracranial and intraspinal neoplasms and unspecified benign/border intracranial and intraspinal neoplasms. # Including carcinomas of the nasopharynx, other sites in the lip, oral cavity and pharynx, and nasal cavity, mid ear, sinuses, larynx and other ill-defined head and neck tumours. ¶ Including carcinomas of other ill-defined sites of the genitourinary tract, gastrointestinal tract, NOS, and adrenocortical carcinomas. ** Including Wilms tumours, neuroblastoma, other paediatric and embryonal tumours, NOS, paraganglioma and glomus tumours, other specified gonadal tumours, myeloma, mast cell, misc lymphoreticular neoplasms, NOS and other specified neoplasms, NOS. †† P-values were obtained from a likelihood ratio test comparing a generalized linear model including the midpoint of the calendar period and a model without calendar period. All models assumed a Poisson distribution for the observed number of deaths. Significance was determined if $p < 0.05$.

Table S5. Absolute numbers of cancer-specific mortality (ICD-10 code C00-C97) cases among adolescents and young adults (AYAs) in the Netherlands between 1990–2016.

Year of Death/Age at Death	Males						Females					
	**15–19**	**20–24**	**25–29**	**30–34**	**35–39**	**15–39**	**15–19**	**20–24**	**25–29**	**30–34**	**35–39**	**15–39**
1990	37	37	49	80	126	329	19	26	52	103	219	419
1991	28	40	69	87	136	360	21	28	61	125	209	444
1992	27	46	38	79	143	333	21	29	60	92	215	417
1993	33	41	50	85	123	332	16	22	61	125	187	411
1994	28	55	40	71	132	326	19	19	50	107	219	414
1995	22	42	46	88	130	328	21	31	44	88	232	416
1996	31	45	51	89	129	345	21	26	45	104	230	426
1997	17	38	37	93	125	310	20	23	38	112	202	395
1998	21	39	51	83	113	307	14	22	38	93	207	374
1999	35	27	54	73	149	338	6	20	40	104	233	403
2000	23	36	55	81	135	330	17	27	38	100	197	379
2001	27	35	30	77	152	321	16	13	39	92	218	378
2002	26	31	41	90	131	319	21	23	47	112	202	405
2003	22	29	45	70	124	290	13	19	48	96	176	352
2004	30	26	42	79	130	307	10	14	34	97	192	347
2005	29	23	42	62	124	280	14	23	38	83	199	357
2006	24	22	32	54	125	257	11	12	39	79	173	314
2007	18	33	34	61	108	254	13	24	34	80	174	325
2008	21	27	37	57	130	272	16	15	40	65	160	296
2009	19	28	46	47	93	233	12	21	38	64	158	293
2010	12	27	34	42	122	237	15	14	30	62	163	284
2011	21	24	23	54	108	230	14	15	35	65	141	270
2012	16	24	31	60	83	214	16	16	28	71	117	248
2013	19	15	32	48	79	193	15	22	23	67	120	247
2014	15	17	33	49	88	202	18	13	36	46	107	220
2015	22	22	29	40	84	197	8	8	27	59	123	225
2016	14	18	24	43	72	171	13	14	32	74	124	257
Total	637	847	1095	1842	3194	7615	420	539	1095	2365	4897	9316
